# Pilot clinical and pharmacokinetic study of Δ^9^-Tetrahydrocannabinol (THC)/Cannabidiol (CBD) nanoparticle oro-buccal spray in patients with advanced cancer experiencing uncontrolled pain

**DOI:** 10.1371/journal.pone.0270543

**Published:** 2022-10-14

**Authors:** Stephen Clarke, Belinda E. Butcher, Andrew J. McLachlan, Jeremy D. Henson, David Rutolo, Sean Hall, Luis Vitetta

**Affiliations:** 1 Royal North Shore Hospital, St Leonard’s, New South Wales, Australia; 2 Faculty of Medicine and Health, The University of Sydney, Camperdown, New South Wales, Australia; 3 WriteSource Medical Pty Ltd., Lane Cove, New South Wales, Australia; 4 School of Medical Sciences, University of New South Wales (UNSW), Sydney, New South Wales, Australia; 5 Sydney Pharmacy School, The University of Sydney, Camperdown, New South Wales, Australia; 6 Faculty of Medicine, Prince of Wales Clinical School, University of New South Wales (UNSW), Sydney, New South Wales, Australia; 7 Medlab Clinical, Alexandria, New South Wales, Australia; Duta Wacana Christian University School of Medicine / Bethesda Hospital, INDONESIA

## Abstract

This pilot study aimed to assess the safety, tolerability, pharmacokinetics and exploratory analgesic effect of a novel water-soluble oro-buccal nanoparticle spray of a cannabis-based medicine (MDCNS-01) in patients with advanced incurable malignancy with unrelieved pain from opioid analgesic. The study was a non-blinded single arm 2 stage study. Stage I was a single escalating dose (n = 5) [2.5 mg Δ9-THC and 2.5 mg CBD) versus a 3-fold escalated dose. Stage II was an up-titrated dose in patients with advanced cancers and intractable pain (n = 25). During Stage I with an increased cannabis-based medicine dose, maximum observed plasma concentrations of cannabinoids were dose dependant. The water-soluble formulation in the current study resulted in a higher median (min, max) systemic exposure of Δ9-THC than CBD (AUC from 2.5 mg each of Δ9-THC and CBD, was 1.71 ng mL.h^−1^ (1.1, 6.6) and 0.65 ng mL.h^−1^ (0.49, 4.1), respectively). During stage II a subgroup of patients diagnosed with breast and prostate cancers with bone metastases, had the highest mean pain score improvement from baseline of 40% (unadjusted) and 33% (adjusted for rescue medication use). For all patients the most reported adverse events were mild or moderate drowsiness affecting 11 (44%) and 4 (6%) patients, respectively, and nausea and vomiting that affected 18 (72%) patients. The water-soluble cannabis-based medicine provided acceptable bioavailability for Δ9-THC/CBD, appeared safe and tolerable in advanced incurable cancers with uncontrolled pain with preliminary evidence of analgesic efficacy.

## Introduction

Relief from chronic pain (cancer or non-cancer related) is a common condition cited by patients for the medical use of cannabis [1,2]. A recent systematic review and meta-analysis of randomized controlled trials (RCTs) suggests that cannabis-based-medicines could be effective for chronic pain treatment and primarily for neuropathic pain [3]. Others have reported that cannabinoid-based pharmacotherapies may serve as effective replacement/adjunctive analgesic options [4].

Most of the studies that have investigated cannabis for analgesia have concentrated on those with neuropathic pain. For example, low dose Δ9-THC (1.29%) as vaporized cannabis relieved central or peripheral neuropathic pain that was resistant to standard treatments compared to placebo [5]. Studies with oral / oromucosal routes of administration of cannabis as herbal crude or dry leaf cannabis extracts or synthetics of THC (dronabinol, nabilone) and plant-derived extracts of THC/CBD oromucosal spray (nabiximols) formulations have also shown efficacy in chronic neuropathic pain [2]. The number needed to benefit (NNTB) for patients taking medicinal cannabis for chronic neuropathic pain was estimated in a Cochrane review as 11 to 20 (95% CIs 7–100), although clinical study sample sizes were small and lacking high-level evidence [2]. All cannabis-based-medicines pooled together were also better than placebo in reducing sleep problems and improving psychological distress and health related quality of life [2]. Medicinal cannabis may also be effective for other types of pain. Studies of smoked cannabis in postsurgical or post-traumatic pain [6] and in painful human immunodeficiency virus (HIV)-associated neuropathy [7–9] have reported efficacy over placebo in relieving pain, as well as being well tolerated [9].

Although the effectiveness of medicinal cannabis for chronic pain has been established [10], all five previous clinical trials of medicinal cannabis for cancer pain (as reviewed by Boland and colleagues [11]) have demonstrated considerable variability in response. Oral administration of cannabis-based medicines leads to highly variable systemic concentrations of pharmacologically active constituents leading to slow and erratic an onset of analgesia [12]. Inhaled cannabis requires frequent dosing to maintain analgesic effect as THC’s half-life is less than 20 minutes [12,13]. Furthermore, the high THC blood concentration (20- to 30-fold higher C_max_) after inhalation is associated with treatment limiting acute adverse effects and long-term damage can also occur from toxic chemicals associated with smoking or vaporizing [12] (high temperatures involved with vaporized cannabis can oxidize the medicine and excipients) [14].

The unsatisfactory nature of oral and inhaled administration routes for medicinal cannabis has led to additional proposed routes of administration such as transdermal and intranasal modes of delivery [15]. Notwithstanding their lipophilic nature, cannabinoids show promise as highly regulated prescribed medicines [15]. We have previously investigated the nanoparticle delivery platform (NanoCelle^™^) in a comparative clinical investigation that demonstrated that an active pharmaceutical ingredient could be safely and efficaciously delivered via the oro-buccal route [16]. Furthermore, in an additional pilot safety tolerability PK study with a CBD-dominant nanoparticle formulation we reported a safe and tolerable primary outcomes when administered to healthy individuals [17]. NanoCelle^™^ is an innovative nanoparticle delivery method for mucous membrane absorption of small lipid soluble molecules such as THC and CBD. The aim of this study was to investigate the safety, tolerability, and preliminary efficacy in a two-dose approach pharmacokinetic investigation of an oro-buccal water-soluble nanosized delivered cannabis-based medicine containing an equal mixture of THC/CBD in advanced cancer outpatients with inadequately controlled pain who self-administered the NanoCelle cannabis-based medicine (MDCNB-01).

## Material and methods

### Study design

This present study was a pilot preliminary safety, tolerability, and PK exploratory endpoint to obtain a possible signal for efficacy. The one compartment PK model was employed in this analysis to characterise the cannabinoids (i.e., CBD, THC) concentration-time data in this study. This PK model represents the simplest approach to describe the PK parameters for CBD and THC, making few assumptions and supported by the blood sampling strategy used in the study. This simple PK model only assumes that CBD and THC are absorbed and eliminated from the body by first-order processes allowing the determination of PK parameters when delivered as an oro-buccal administered cannabis-based medicine. We appreciate that other published studies have employed 2 and 3 compartment PK models to analyse CBD and THC concentration-time data. However, the extensive blood sampling strategies that allow the characteristics of more complex PK models for CBD and THC disposition were not feasible or appropriate in this patient population undergoing treatment for advanced cancer. Using the one compartment PK model allows for robust and relevant estimates of the major PK parameters such as clearance, volume of distribution and half-life.

The study consisted of two stages, namely, Stage I which was a two-day single ascending dose (SAD) and a multiple ascending dose (MAD) simple pharmacokinetic investigation of the nanoparticle Δ9-THC/CBD formulation. Five participants diagnosed with advanced incurable cancer as confirmed by the treating clinician and with controlled pain was deemed suitable for a preliminary PK assessment of the cannabis-based medicine. On day 1 all participants were administered 2.5 mg THC and 2.5 mg of CBD in 300 μL (two actuations of the pump) to the oro-buccal mucosa. On day 2 all subjects administered 7.5 mg of Δ9-THC and 7.5 mg of CBD in 900 μL (six actuations of the pump [3 doses]). Blood samples were collected at 0, 30, 60, 90, 120, 150, 180, 240, 360 min and 24 h after dose administration via indwelling cannula.

Stage II was the up-titrated pilot study with 25 eligible participants diagnosed with advanced incurable cancer and uncontrolled pain as confirmed by the treating clinician while currently being treated with opioid analgesics. The stages of malignancy were verified but related on investigator assessment. Eligible participants were invited to participate over the three phases in a 30-day period. The three phases in Stage II were divided into a dose escalation phase over days 1 to 9; a treatment phase over days 10 to 15; and a follow-up phase over days 16 to 30. Prospective participants for all Stage II phases of this pilot study attended the Royal North Shore Hospital Medical Oncology unit for follow-up with the study investigator.

**Fig 1:**
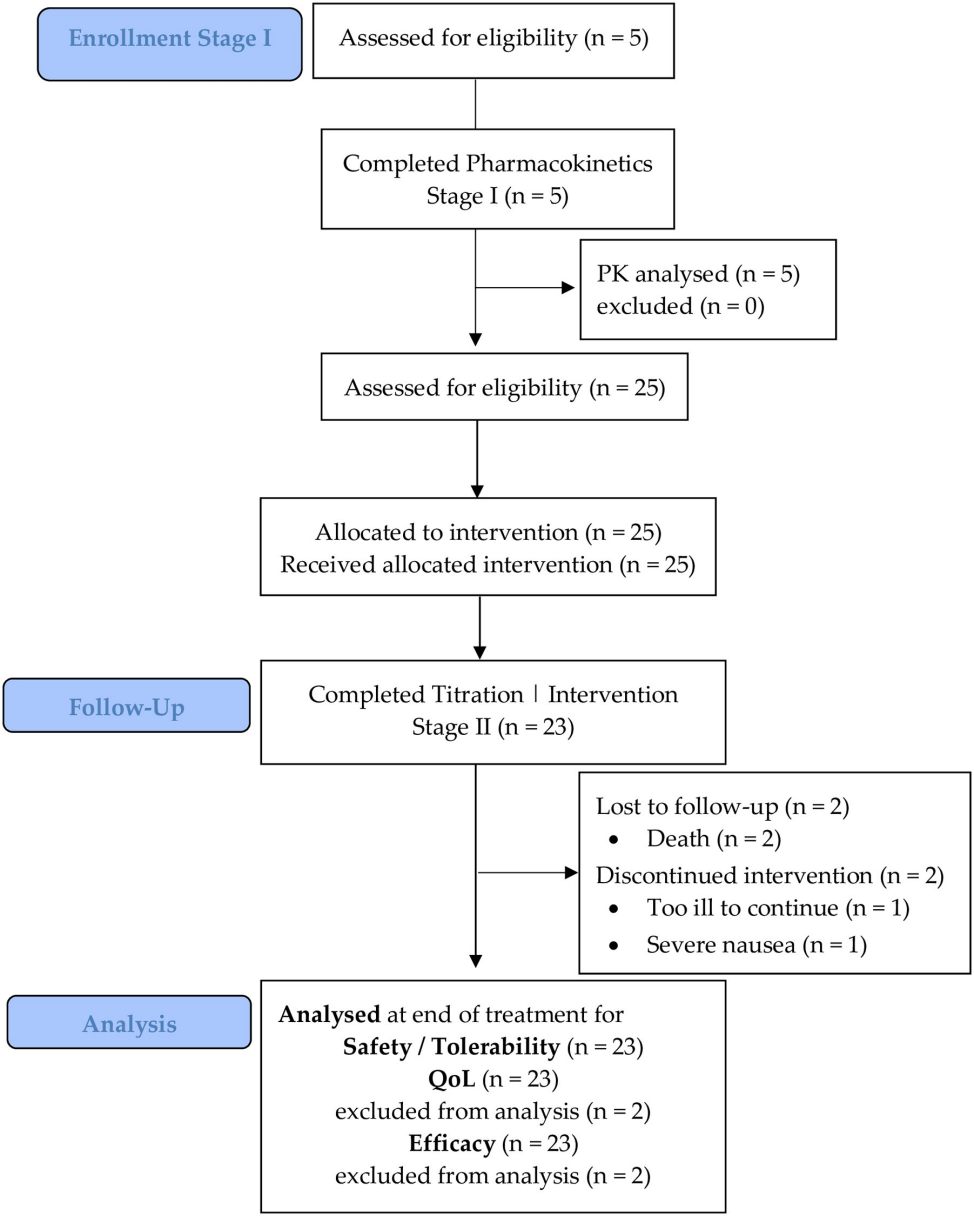
CONSORT diagram for Single Ascending (SAD) and Multiple Ascending Dose (MAD) Stages I and II pilot study. In Stage I (pharmacokinetics) eligible patients administered one dose (2 sprays) on day 1 and three doses (6 sprays) on day 2. In Stage II after baseline assessments, patients commenced with one spray of the cannabis-based medicine (1.25 mg each of Δ9-THC and CBD in 0.15 mL) every 4 to 8 hours while awake for 3 days, then dose escalated to two sprays every 4–8 hours while awake for 3 days, and finally dose escalated to three sprays every 4–8 hours while awake for 3 days.

Screening visits occurred −2 to 0 days prior to commencing administration of the THC/CBD water soluble nanoparticle oro-buccal spray (see Supplemental digital content). All participants commenced dose escalation; only 23 patients commenced the treatment phase; and 22 patients the follow-up phase of Stage II (Fig 1). Hence, only n = 22 of 25 patients completed all phases of the study’s Stage II.

This pilot clinical study was approved by the North Sydney Local Health District Human Research Ethics Committee and registered with the Australian New Zealand Clinical Trial Registry (number ACTRN12617001480370).

During Stage I of the study patients administered one dose and then three doses only on consecutive days for the PK arm of the study. During Stage II of the dose escalation phase patients administered to the oral cavity (interchanging to the inside of either cheek) one, two or three actuations (1 actuation = 1 spray) every 4–8 hours (unless asleep) for six days. Patients that progressed to the treatment phase of Stage II administered one, two or three doses every 4–8 hours (1 dose = 2 sprays) unless asleep that was dependent on the safe and tolerable dose administered in the previous dose escalation phase of the study, as directed by the treating clinician.

In the follow-up phase, all patients were observed for the next 15 days after treatment cessation. Subsequently, all patients who completed the study were provided with compassionate access to the ongoing cannabis-based medicine. Plasma samples whether during Stage I (on Days 1 and 2 at 0, 30, 60, 90, 120, 150, 180, 240, 360 min and 24 h) or during Stage II were collected in-hospital prior to the first morning’s administration of the cannabis-based medicine on days 1, 4, 7, 10, 13, 16 and 30.

### Oro-buccal (inside of the cheek) dosing

Medlab Clinical formulated the investigational product (MDCNB-01) which was manufactured by PCI Pharma Services (Melbourne, Australia) in a GMP accredited facility. PCI Pharma also conducted the filling of the vials containing the drug substance, labelling, and packaging and quality assurance release. The formulation MDCNB-01 was derived from plant material. The formulation consisted of 97.5% THC and CBD with the remaining 2.5% consisting of cannabidiolic acid, cannabigerol, cannabinol and cannabichromene. The Δ9-THC and CBD NanoCelle^™^ (MDCNS-01) is a water-soluble submicron particle suspension light amber in color that also contains glycerol, peppermint oil flavor and a non-ionic oil-in-water solubilizer and emulsifying agent that is administered to the oro-buccal mucosa through a sealed pump action device that produced a fine mist spray. One actuation of the pump in this study administered 1.25 mg Δ9-THC and 1.25 mg CBD in a 150 μL volume.

### Sample analysis

The cannabinoid concentrations were measured using a validated assay that was co-developed with Medlab Clinical and that was conducted by Agilex Biolabs (previously known as CPR pharma services), a National Association of Testing Authorities (NATA) accredited facility based in Adelaide, Australia. Agilex Biolabs co-developed and validated the LC-MS/MS assay method. Blood samples from all eligible participants were assayed for Δ9-THC, CBN, CBD, 11-hydroxy-THC (OH-THC) and 11-nor-9-carboxy-THC (COOH-THC). Determination of cannabinoids CBD, CBN, Δ9-THC, THC metabolites 11-hydroxy-THC (11-OH-THC) and 11-nor-9-carboxy-THC (COOH-THC) and their respective deuterated Internal Standards were analysed in human plasma (100 μL) prepared using protein precipitation and solid phase extraction (using acidified acetonitrile and micro-elution plates). The analytes were separated by high performance liquid chromatography (HPLC) on a Phenomenex Kinetex Biphenyl column, and the eluates monitored by a QTRAP5500 tandem mass spectrometry (MS/MS) detector in negative MRM mode. The data were acquired an Analyst^®^ (Sciex) system linked directly to the QTRAP5500 MS/MS detector and then processed in Watson LIMS^™^ (Thermo Scientific), where applicable. The calibration range was from 0.100 to 10.0 ng/mL for CBD, CBN, Δ9-THC and OHTHC and from 1.00 to 100 ng/mL for COOHTHC, using 100 μL of plasma and has a run time of approximately 8 minutes (as duplicate samples).

Laboratory assessments of the cannabinoids showed that there was no significant interference at the retention time of the internal standard (IS). Also, there was no significant interference at the retention time of the other analytes for each individual analyte injected at PS8 concentration without IS added (except for analyte CBN where the percentage interference was approximately 29.0% of the mean CBN Lower Level of Quantification response when Δ9-THC was injected at Upper Level of Quantification level of 10.0 ng/mL). There were 5 repetitions performed on the same day. Coefficient of determination for curves run during validation had a linearity of >99%. Moreover, inter- and intra-assay precision had a mean coefficient of variation of approximately 3% (n = 18) and inter- and intra-assay accuracy had a mean % bias of approximately 3% (n = 18). Furthermore, there were no significant effects observed for any of the analytes in six individual lots plus in lipemic and hemolysed sources. The assay limit of detection for all metabolites was 0.1 ng/mL.

### Inclusion and exclusion criteria

Patients were eligible for inclusion in **Stage I** if they: **i)** were aged greater than 18 years; **ii)** gave informed consent and agreed to comply with the study procedures (inlcuding a THC urine screen at baseline); **iii)** had been diagnosed with an advanced incurable malignancy; **iv)** were willing to abstain from using other cannabis-based medicines/recreational cannabis; **v)** agreed, where applicable, to use an effective form of birth control; **vi)** consented to baseline test for pregnancy; **vii)** consented to baseline tests for recent cannabis use; **viii)** agreed not to drive a car or other motor vehicle or operate any type of heavy machinery for 72 hours after the last dose of study medication.

Patients were eligible to be included in **Stage II** if they: **ix)** reported experiencing moderate to severe pain; **x)** were currently receiving strong opioid analgesics for at least one week to relieve pain associated with incurable malignancy (minimum one-week prior use of opioid treatment is sufficient duration because it would represent established opioid treatment and most patients would have developed tolerance after one week, especially with multiple daily doses totalling > 60 mg of oral morphine equivalent) and/or patient is on a low dose opioid regimen and still experiencing pain and is unable to increase the opioid dose due to poor tolerance as confirmed by the treating clinician; **xi)** reported pain severity greater than four on a 0–10 Numerical Rating Scale (NRS) assessment tool; xii) patients agreed to not partake in any other interventional clinical treatments other than the ones that the treating clinical team were already aware of for the duration of the study.”

Patients were **excluded** from participation if they met any of the following: **i)** demonstrated cognitive impairment or intellectual disability; **ii)** had a history of primary psychotic disorder, bipolar affective disorder, bipolar disorder with psychotic features, depressive disorder with psychotic features, borderline personality disorder, antisocial personality disorder, or a positive family history (first degree relative) of psychotic disorder or bipolar affective disorder; **iii)** had any history of allergic or hypersensitivity reaction to any herbal product, including cannabinoids; **iv)** reported a prior sensitivity reaction to an oro-buccal administered medicine or supplement (e.g., liposomes); **v)** had undergone radiotherapy to the mouth or oral cavity; **vi)** had significant intercurrent medical illnesses that the PI assessed to make them unsafe to be enrolled; especially a history of epilepsy (or a previous history of seizures), or clinically significant hepatic or renal impairment; **vii)** uncontrolled brain metastases; **viii)** pregnant or breast feeding; **ix)** had received epidural analgesia within 48 hours of the baseline assessment; **x)** had received radiotherapy within two weeks of the initial baseline assessment; **xi)** were currently receiving levodopa, sildenafil (or any other PDE5 inhibitors), anticonvulsants and/or cannabinoids, or ketamine; **xii)** participants were ineligible with a presentation of clinically significant liver dysfunction defined by 2.5 times higher abnormal liver function tests (in the absence of hepatic metastasis); and clinically significant kidney dysfunction defined by 1.5 times decreased glomerular filtration rate (GFR of < 50); and relevant to the full blood count a white blood cell count > 1,500 cells / μL and platelets > 100,000 cells / μL.

Eligible participants were carefully instructed (provided with written information) and initially observed by nursing staff on how the cannabis-based medicine spray was to be administered to the oro-buccal area. Patients were also informed that they should not consume food, beverages (for at least 60 minutes before or after using the spray) or speak during the administration process of the cannabis-based medicine.

All participants provided written informed consent and provided a urine sample at baseline for a qualitative THC screening prior to commencing participation into the study. There was no indication from baseline documented patient reports that enrolled participants had previously used recreational cannabis or had self-treated symptoms with cannabis.

Participants were included if they were diagnosed with advanced incurable malignancy with controlled pain (Stage I) and intractable pain unrelieved by prescribed opioid analgesics for Stage II of the study.

### Clinical study endpoints

*Primary Endpoints*: consisted of a clinical assessment of safety and tolerability from recorded adverse events/serious adverse events; quality of life scores as measured with The European Organisation for Research and Treatment of Cancer Quality of Life for Cancer Patients Questionnaire (EORTC QLQ-C30-v3) [18]. For example the minimum threshold for clinical importance for **functioning scales scores** were: physical functioning, 83; role functioning, 58; social functioning, 71; emotional functioning, 71; cognitive functioning, 75. The minimum threshold for **symptom scales scores** were: fatigue, 39; pain, 25; nausea/vomiting, 8. The minimum threshold for **sleep disturbances scores** were: dyspnea, 17; appetite loss, 50; constipation, 50; diarrhea, 17; financial impact, 17. The sensitivities of these thresholds for clinical importance ranged from 0.71 to 0.91 [18]. Patients completed questionnaires independent from the treatment physician. All adverse events including those recorded due to the diagnosed illness and with the short-term use of the cannabis-based medicine were scored as mild, moderate, or severe. As previously reported [19] recording lower (worse case) scores on the EORTC QLQ-C30-v3 at baseline were deemed to be associated with SAEs occurrence during the pilot trial.

Furthermore, pharmacokinetic parameters AUC, C_max_, T_max_ and bioavailability were determined.

*Secondary endpoints*: cannabis treatment analgesic efficacy as assessed by Mean Numerical Pain Rating Scale (NPRS) scores; Morphine Milligram equivalent (MMeq) doses; rescue analgesia (opioid) doses.

### Study safety endpoints

At each visit the incidence and severity of adverse events were recorded. Adverse events were documented as per the criteria used for the AEs as ascertained from the Joint Commission on Accreditation of Healthcare Organizations (JCAHO) Patient Safety Event Taxonomy [20] that facilitated a common approach for patient safety information in this pilot study. Any clinically significant changes in vital signs or physical examination were reported as adverse events. Clinically relevant changes in concomitant medications, e.g., changes in morphine milligram equivalent doses were also recorded (see Supplemental digital content).

### Statistical methods

This study was largely an exploratory pilot study and as such a sample size of 5 participants was considered adequate for a limited assessment of the Stage I single dose PK study with no sample size calculation *a priori*. This was similar in design to a phase I study that assessed the single and multiple dose PKs and safety and tolerability of a mouth spray that administered Δ9-THC/CBD [21]. For Stage II sample size of n = 25 was sufficient to detect a moderate effect size of 0.5 with alpha set at 0.05 (one-tail) and power set at 80%.

Analysis of the primary outcome (safety and tolerability) was descriptive. For Stage I, given the sample size, continuous outcomes were reported as medians and interquartile ranges. Pharmacokinetic analysis was conducted using a one-compartment open pharmacokinetic model with first order absorption and elimination. The area under the curve (AUC) from 0 to 6 h was calculated using the linear trapezoidal rule, the maximum concentrations (C_max_) and the time it occurred (T_max_) were determined by observation of the concentration-time profile curve and the half-life (t½) was calculated from the elimination rate constant (kel) which was estimated from the slope of the terminal portion of the log-concentration-time profile.

The daily pain NPRS score was calculated as the mean of all the daily assessments. The change in mean NPRS pain scores from baseline (all days in run-in period) to the end of treatment was analysed using analysis of covariance (ANCOVA), with baseline pain as a covariate and grouped study and treatment as factors [22]. Change scores were used to reduce the possible impact of regression to the mean. The proportions of responders (patients with ≥ 30% improvement from baseline to end of study NPRS scores) was compared. Use of breakthrough medication (during treatment) was recorded. In addition, the change from baseline in mean number of doses of escape medication was analysed using ANCOVA. The EORTC-QLQ-30 was scored according to its scoring manual [23], and differences over time explored using ANOVA.

Normality was tested using Shapiro-Wilk test of normality, with p-values <0.05 considered a significant deviation from normality. Normally distributed variables are reported as mean (SD), non-parametric variables as median (interquartile range [IQR]). All analyses were conducted in Stata MP V16 for Mac (StataCorp, Texas Station, US).

## Results

Patient demographics and clinical characteristics are presented in Tables 1 and 2. In Stage I five eligible participants with controlled pain and that were diagnosed with an advanced incurable malignancy as documented by the principal investigator of the study were included (Table 1). In Stage II all 25 eligible participants (Table 2) were diagnosed with an advanced incurable malignancy with intractable pain as documented by the principal investigator of the study. There was a wide range of tumor types and sites of metastases (Table 2). There was a mixture of different types of pain, including patients with multiple types of somatic and visceral pain. The most common types of pain diagnosed were neuropathic (11), nociceptive (9) and bone (8) in origin. Furthermore, 8 patients (32%) presented with diagnosed bone metastases causing pain. Eligible participants remained under the supervision of the principal investigator for the duration of the pilot study.

**Table 1:** Participant baseline demographic and clinical characteristics diagnosed with advanced incurable malignancy with controlled pain for Stage I (n = 5 [100%]).

Demographics Stage I	Number n (%)
**Sex**	
Males	2 (40%)
Females	3 (60%)
**Age**	**Median (IQR)**
Years old	62.0 (56.0, 73.0)
**Ethnicity**	
European	5 (100%)
**Cancer Diagnosis**	**Number (%)**
Glioblastoma	1 (20%)
Lung	1 (20%)
Myeloma	1 (20%)
Thyroid	1 (20%)
Prostate	1 (20%)

**Table 2:** Participant baseline demographic and clinical characteristics diagnosed with advanced incurable malignancy with uncontrolled pain for Stage II (n = 25 [100%]).

Demographics Stage II	Number n (%)
**Sex**	
Males	10 (40%)
Females	15 (60%)
**Age**	**Mean (SD)**
Years old	55.9 (11.9)
**Ethnicity**	**Number (%)**
European	20 (80%)
Hispanic/Latino	1 (4%)
East Asian	4 (16%)
**Cancer Diagnosis**	**Number (%)**
Breast	7 (28%)
Non-Small Cell Lung Cancer (NSCLC) Lung	4 (16%)
Gastrointestinal	4(16%)
Hematological	3 (12%)
Pancreas	2(8%)
Ovaries	2(8%)
Melanoma	1(4%)
Central Nervous System	1(4%)
Prostate	1(4%)

### EORTC-QLQ-30

**Fig 2:**
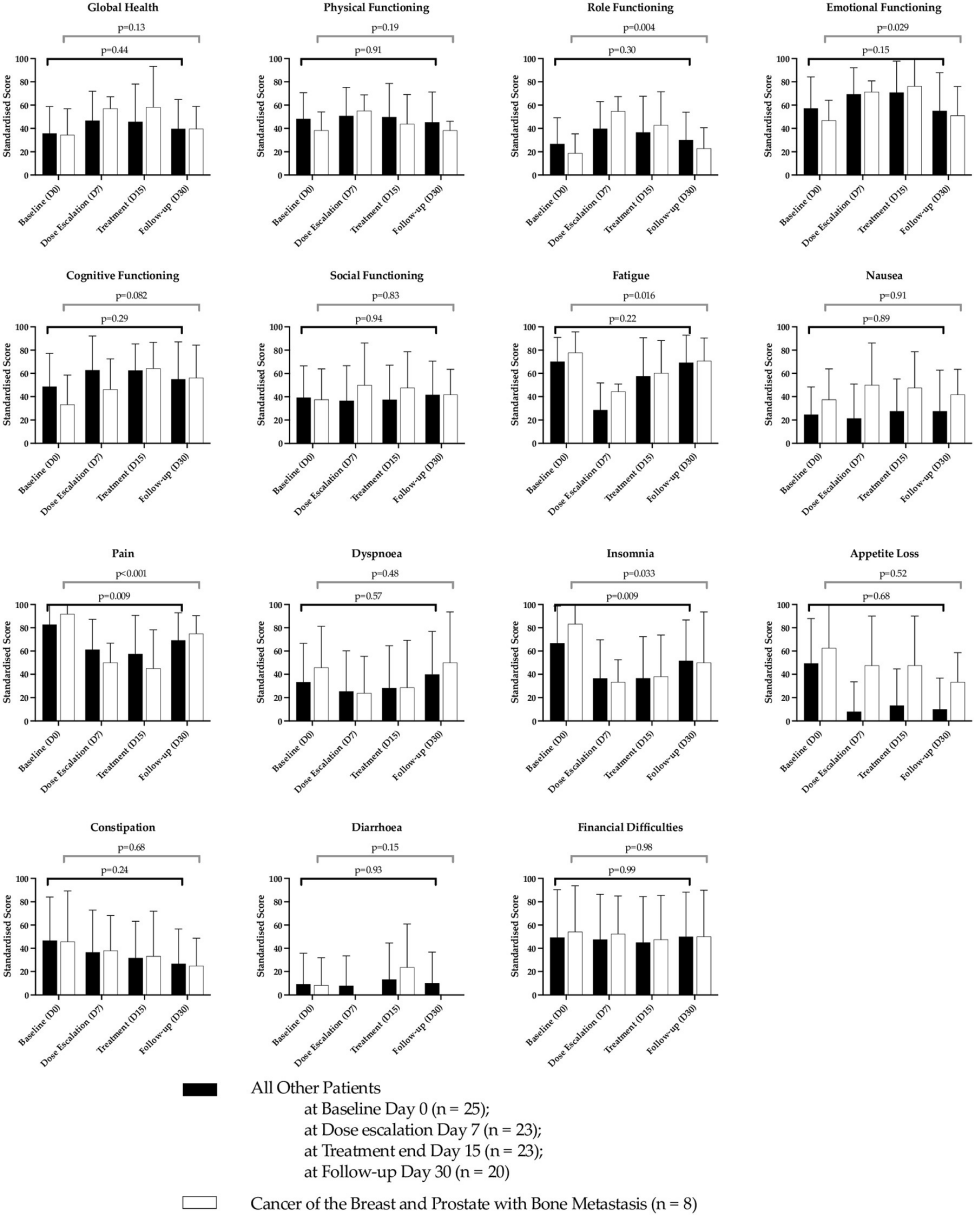
EORTC-QLQ-30 scores relevant to improvement or deterioration of global functioning and symptoms between patients that demonstrated improvement in pain from those patients with breast and prostate cancers and bone metastasis compared to patients from the remainder of the cohort. Error bars represent STD.

The patients completed the questionnaire at the induction visit and subsequent visits to the hospital and the treating clinician was blinded to this process. Given the complex presentation of the cohort of patients under investigation, we report the EORTC-QLQ-30 scores for those patients diagnosed with breast and prostate cancer with metastasis to the bone compared to those patients representing the rest of the cohort (Fig 2).

There was an overall improvement from baseline for global health status; physical functioning; emotional functioning; cognitive functioning; fatigue; pain; dyspnoea and insomnia (Fig 2). The EORTC-QLQ-30 scores established preliminary thresholds for clinical importance in the patients that presented with breast and prostate cancers and diagnosed with bone metastasis as compared to the total cohort of patients in the study.

### Pharmacokinetic parameters

The PK assessment of a single dose of 2.5 mg each of THC and CBD (2 sprays on Day 1) versus 7.5 mg each of Δ9-THC and CBD (six sprays on Day 2) was investigated for all participants in Stage I. The PK parameters calculated for Δ9-THC, CBD and Δ9-THC metabolites, 11-OH-THC and COOH-THC, are presented in Table 3. There was a rapid uptake of Δ9-THC and CBD, as indicated by detectable plasma concentrations at the first 30 minute time point following oro-buccal administration (Fig 2) and cannabinoids having a median t_max_ of 0.75 hours (Table 3). Single doses of Δ9-THC and CBD were rapidly eliminated, with 2.5 mg of Δ9-THC and CBD eliminated in just under 1 hour and 45 minutes, respectively, and approximately twice that duration for the higher 7.5 mg dose (Table 4). The plasma concentration of the main active metabolite of Δ9-THC, OH-THC, peaked soon after THC (0.12 and 0.4 hours after Δ9-THC for the low and high dose, respectively) and persisted for approximately three-fold longer (OH-THC t_1/2_ was 3.4 and 5.0 hours for the low and high dose, respectively).

**Table 3:** Participant baseline clinical characteristics for Stage II (n = 25).

Type of Primary Cancer*	Number n (%)	Type of pain** (n)
Breast + bone metastasis	5 (20%)	Neuropathic (2)
		Neuropathic, Nociceptive (1)
		Bone pain (2)
Breast + lung + bone metastasis	1 (4%)	Neuropathic, Bone pain
Breast + liver + bone metastasis	1 (4%)	Neuropathic, Bone pain
NSCLC + brain + bone metastasis	4 (16%)	Neuropathic, Visceral, Neuropathic, Bone pain
NSCLC + bone metastasis		Neuropathic, Nociceptive, Somatic, Bone pain
NSCLC + adrenal gland + liver metastasis		Neuropathic, Nociceptive, Somatic, Bone pain
Lung + bone metastasis		Nociceptive, Visceral, Bone pain
Gastrointestinal	4 (16%)	
• Oropharyngeal + lung metastasis + lymph nodes		Neuropathic
• Oesophageal + liver + lung metastasis		Nociceptive, Visceral, Neuropathic
• Large bowel + peritoneum + lung + bone metastasis		Neuropathic, Nociceptive, Visceral
• Appendix mucinous + peritoneum metastasis		Neuropathic
Hematological	3 (12%)	
• Diffuse large cell lymphoma		Neuropathic
• CLL + SCC scalp metastasis		Neuropathic
• Multiple Myeloma + BPH		Chronic Bone pain
Pancreas + lung + liver + adrenal gland	1 (4%)	Nociceptive, Visceral, Neuropathic
Pancreas + left adrenal + peritoneum + mediastinum + para-aortic and mesenteric lymph node metastasis	1 (4%)	Neuropathic, Visceral
Ovaries + liver + lung metastasis	1 (4%)	Nociceptive, Visceral, Bone pain
Ovaries + breast + groin + abdominal lymph nodes metastasis	1 (4%)	Nociceptive, Visceral
Melanoma + bone metastasis	1 (4%)	Neuropathic
Central Nervous System	1 (4%)	Neuropathic
Prostate + bone metastasis	1 (4%)	Neuropathic

*All eligible enrolled patients were diagnosed with advanced incurable malignancy with intractable pain unrelieved by opioids.

**Some patients presented with more than one type of pain.

NSCLC = Non-Small Cell Lung Cancer; CLL = Chronic Lymphocytic Leukemia; SCC = Squamous Cell Carcinoma; BPH = Benign Prostatic Hypertrophy.

**Table 4:** Summary of Stage I two dose-approach of n = 5 (100%) (one dose escalating to three doses) cannabinoid pharmacokinetic parameters.

Parameter	Day 1 (n = 5) 2.5 mg Δ9-THC+ 2.5 mg CBD Median (Min, Max)	Day 2 (n = 5) 7.5 mg Δ9-THC+ 7.5 mg CBD Median (Min, Max)
**Δ9THC** ^⊥^		
AUC_(0-t_) ng mL.h^−1^	1.71 (1.11, 6.61)*	8.26 (2.67, 11.72)
C_max_ ng mL^−1^	1.31 (0.76, 2.94)*	2.35 (1.09, 3.19)
t_max_ hours	0.75 (0.5, 1.5)*	1.00 (0.5, 2.0)
t_1/2_ hours	0.94 (0.75, 1.14)*	1.39 (1.30, 2.88)
**CBD**		
AUC_(0-t)_ ng mL.h^−1^	0.65 (0.49, 4.06)*	5.96 (1.51, 12.15)*
C_max_ ng mL^−1^	0.58 (0.48, 2.45)*	1.55 (0.62, 2.25)
t_max_ hours	0.75 (0.5, 1.5)*	1.00 (0.5, 2.0)
t_1/2_ hours	0.72 (0.57, 0.86)*	1.53 (1.16, 7.06)
**11-OH-THC**		
AUC_(0-t)_ ng mL.h^−1^	3.10 (2.17, 49.37)	17.2 (7.91, 99.13)
C_max_ ng mL^−1^	2.06 (0.29, 13.8)	3.74 (1.06, 20.4)
t_max_ hours	1.00 (0.5, 1.5)	1.50 (0.5, 2.0)
t_1/2_ hours	4.05 (1.19, 5.23)	5.31 (1.60, 8.02)
**COOH-THC**		
AUC_(0-t)_ ng mL.h^−1^	126.32 (34.29, 251.29)*	223.39 (162.57, 1172.98)
C_max_ ng mL^−1^	13.70 (6.62, 25.40)*	26.80 (13.1, 96.0)
t_max_ hours	1.25 (0.5, 2.0)*	2.5 (1.5, 3.0)
t_1/2_ hours	10.94 (2.34, 12.33)*	10.09 (7.41, 19.23)

^⊥^ Δ9THC = delta-9-tetrahydrocannabinol; CBD = Cannabidiol; 11-OH-THC = 11-hydroxy-tetrahydrocannabinol; COOH-THC = carboxy-tetrahydrocannabinol, AUC = area under the plasma concentration versus time curve, from time zero to the last measurable concentration at t = 6 hr; Cmax = maximum measured plasma concentration over the time span specified; Tmax = time of maximum measured plasma concentration; t1/2 = time required for the concentration of the drug to halve;

* for n = 4 patients only due to one sample not evaluable.

For the Stage I part of this pilot clinical study, participants remained under clinical supervision in hospital for 48 hours to carefully assess safety and tolerability of the cannabis-based medicine administered and for blood sample collections. No within-participant comparisons were made between day 1 and day 2 pharmacokinetic parameters. Furthermore, during Stage I of this preliminary study, additional peaks in the plasma cannabinoid concentration-time graphs indicative of cannabis-based medicine swallowing were not observed.

**Fig 3:**
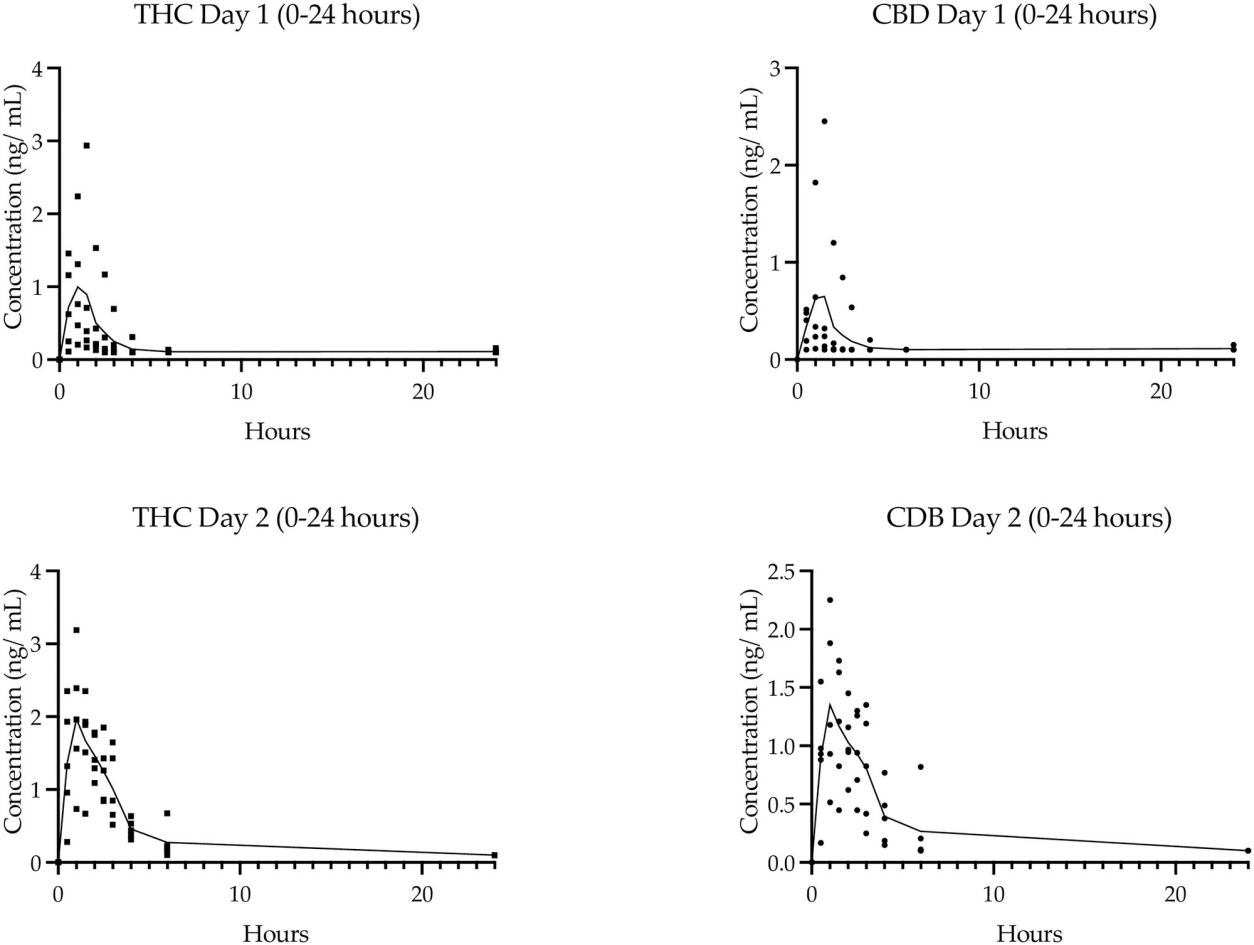
PK of Stage I SAD. Individual and median plasma concentration over time for Δ9-THC (squares) and CBD (circles) on Day 1 (single dose of 2.5 mg Δ9-THC/2.5 mg CBD) and Day 2 (3-fold dose of 7.5 mg Δ9-THC/7.5 mg CBD).

Median plasma concentrations of Δ9-THC, CBD, OH-THC and COOH-THC were dose dependant, with a six-fold high dose providing a 4.8-, 9.2–5.5- and 1.8-fold higher area under the curve (AUC) and a 1.8-, 2.7-, 1.8- and 1.9-fold higher C_max_, respectively (Table 3). The single ascending dose PK analysis of this study showed that C_max_ and bioavailability was always higher for Δ9-THC than for CBD. For the 2.5 mg Δ9-THC/2.5 mg CBD dose, the median C_max_ of Δ9-THC was 1.31 ng/mL, 2.3-fold higher than the 0.58 ng/mL for CBD; and the median AUC_(0-t)_ of Δ9-THC was 1.71 hr.ng/mL, 2.5-fold higher than the 0.65 hr.ng/mL for CBD (Table 3). Furthermore, as the concentration of the drug was increased from 2 sprays on day 1 to 6 sprays on day 2, there was an increased change in the plasma concentrations of Δ9-THC and CBD that demonstrated non-linear PK behaviour Fig 3).

In the Stage II of the pilot study plasma samples were also collected prior to the first morning administration of the cannabis-based medicine on days 1, 4, 7, 10, 13, 16 and 30.

### Adverse events (AEs) and serious adverse events (SAEs)

Adverse events were reported by patients daily to the clinical staff that were conducting the pilot clinical trial. All causally related AE that occurred during the treatment periods over 15 days (treatment-emergent adverse events) and their frequency are presented in Table 5 [20]. Some patients experienced multiple AEs. The most common treatment emergent AE was drowsiness (mild, moderate, and severe in 68%, 44% and 16%, respectively). Fatigue was also common experienced as mild in 4%, moderate in 20% and severe in 12% of patients. Vomiting was also reported as mild in 20%, moderate in 4% and severe in 12% of patients respectively. No serious adverse events were reported. The cannabis-based medicine was considered safe during a short-term exposure in this cohort of patients with incurable malignancies. Furthermore, by continuing to administer the cannabis-based medicine the drug was also deemed tolerated by the patients, even after associated side effects such as drowsiness, fogginess, nausea and dry mouth were noted to continue during the treatment phase of the study.

**Table 5:** Treatment emergent adverse events affecting one or more participants during the MAD Stage II of the pilot trial (n = 25).

Adverse Event Description	Mild n (%)	Moderate n (%)	Severe n (%)
Auditory hallucination	1 (4%)	-	-
Burning throat	1 (4%)	-	-
Constipation	2 (8%)	1 (4%)	-
Dizziness	10 (40%)	1 (4%)	-
Drowsiness	17 (68%)	11 (44%)	4 (16%)
Dry mouth	1 (4%)	3 (12%)	2 (8%)
Fatigue	1 (4%)	5 (20%)	3 (12%)
Fogginess	5 (20%)	2 (8%)	1(4%)
Hallucinations	-	2 (8%)	-
Impaired Concentration	1 (4%)	-	-
Lethargy	-	1 (4%)	1 (4%)
Nausea	9* (36%)	5 (20%)	1 (4%)
Nightmare	1 (4%)	-	-
Numbness bottom lip	1 (4%)	-	
Pain crisis post coming off IP*	-	-	1 (4%)
Restless at night	-	1 (4%)	-
Vivid dreams	1 (4%)	-	-
Vomiting	5 (20%)	1 (4%)	3 (12%)

*One patient developed nausea after administering one dose of the cannabis-based medicine (2 sprays) as a standardized AE [20].

In 9 of 24 patients (37.5%), reported emesis that was associated with nausea. In one patient nausea and vomiting were reported as persistent that subsided on interrupting the administration of the cannabis-based medicine. The last administered dose in this patient occurred on day 9 (first dose of the day) of the dose escalation phase of Stage II. In this patient the cannabis-based medicine was not tolerated at a dose frequency of 8 doses per day. The patient discontinued administering the cannabis-based medicine for the rest of the dose escalation period and the treatment phase.

There were no serious adverse events (e.g., life threatening, hospitalisations requiring medical or surgical interventions, disability temporary or permanent, death) associated with the administration of the cannabis-based investigational medicine.

### Pain medications administered

**Fig 4:**
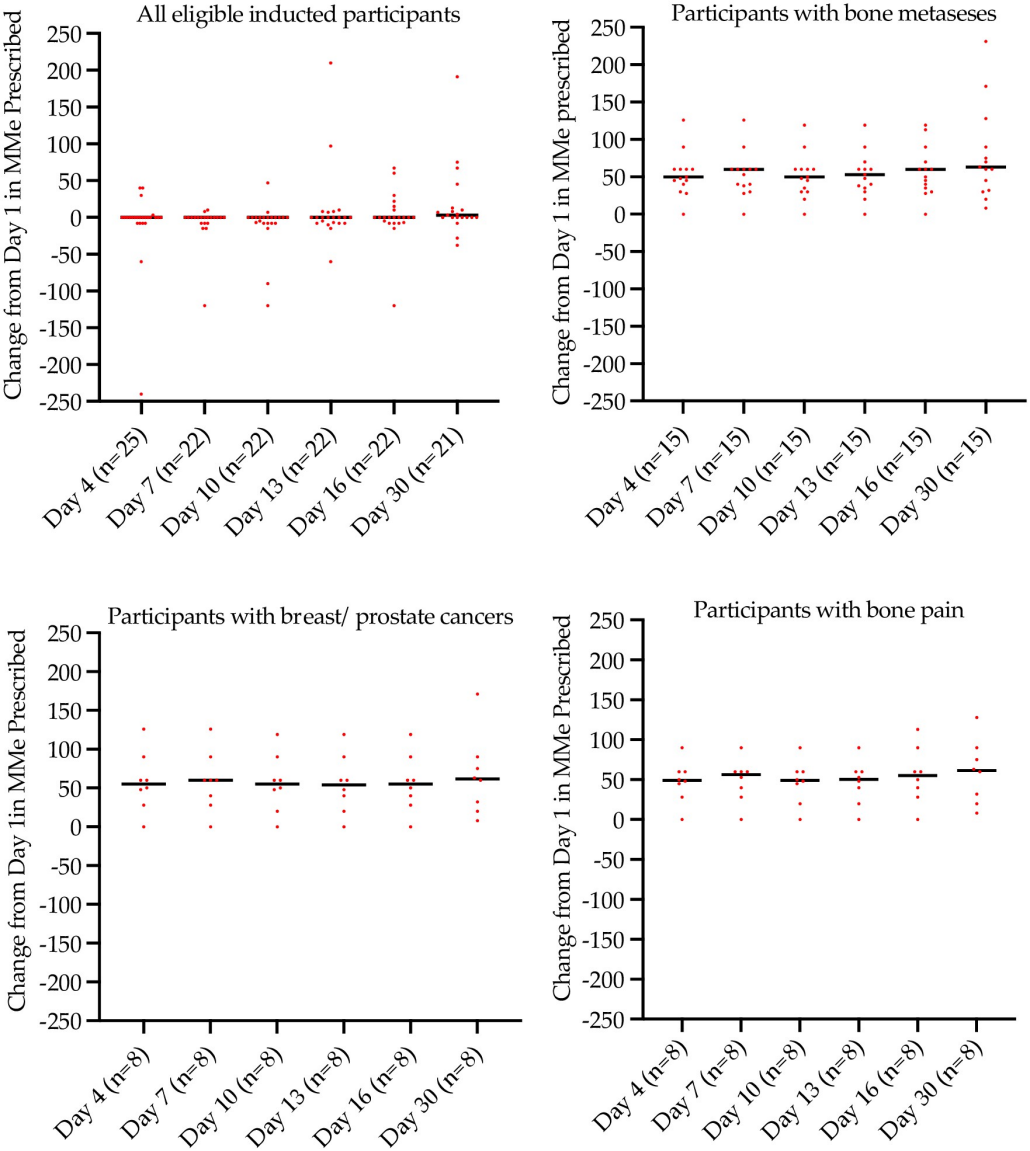
Change in morphine milligram equivalent dose prescribed from day 1. Note, one patient reported highly variable changes in MMeq over the time course of the study (Day 4, -240 MMeq; Day 7–330 MMeq; Day 10–90 MMeq; Day 13 +210 MMeq; Day 16 +30 MMeq; Day 30 +1170 MMeq. Day 7 and Day 30 changes are not depicted on this figure for this patient).

The cohort in this study consisted of patients diagnosed with advanced incurable cancers with uncontrolled pain unresponsive to opioids prescribed for the management of pain. Fig 3 shows the oral morphine milligram equivalent (MMeq) dose recorded as the study progressed from day 1 to day 30. During study progression from day 1 to day 30 all patients (n = 22 of 25 that completed Stage II) as a group recorded an increase in MMeq with a median (IQR) on day 1 (baseline) of 60 (45, 170) mg, on day 16 (end of intervention) of 60 (40, 113) mg and on day 30 (end of follow-up) of 63 (32, 128) mg, respectively (Fig 4).

The most frequently prescribed rescue opioids were oxycodone, hydromorphone and morphine. As a group, patients diagnosed with metastatic breast and prostate cancer reported less prescribed rescue medications for the management of pain.

Furthermore, other medications that were administered for pain management to participants enrolled in Stage II of the pilot study included pregabalin for 2 breast cancer patients (dose: 25 mg daily and 75 mg twice daily); one prostate cancer patient (dose: 25 mg daily); one CLL patient (dose: 75 mg daily); one patient with pancreatic cancer (dose: 25 mg daily); one ovarian cancer patient (dose: 50 mg twice daily); and one patient with NSCLC (dose: 75 mg *mane* and 150 mg *nocte*).

**Fig 5:**
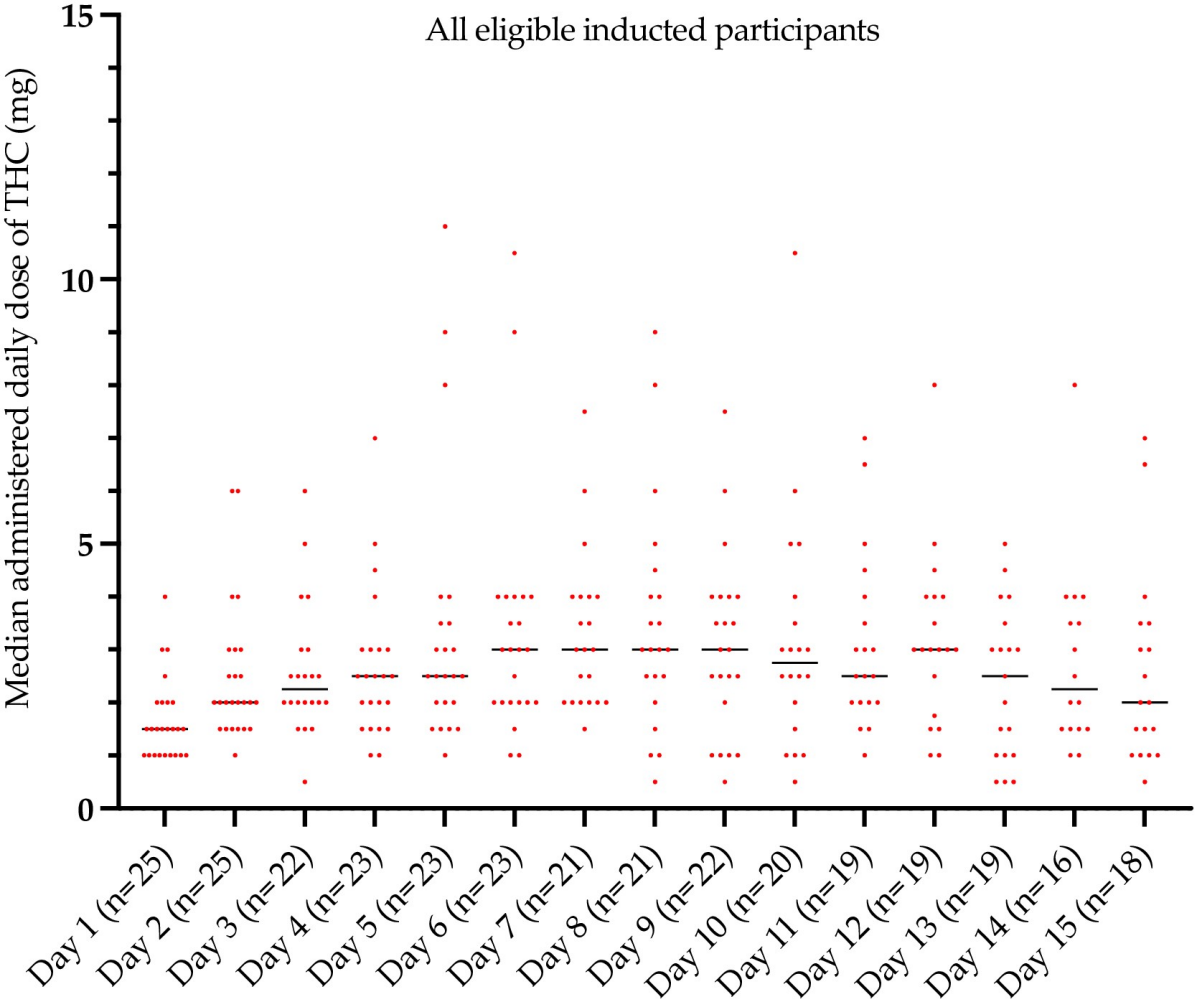
Average daily dose of daily Δ9-THC doses administered every 4–8 hours unless asleep from day 1 to day 15. [Day 1–3, Day 4–6, Day 7–9 = dose escalation phase; Day 10–15 = treatment phase]. Two and a half times the median amount of Δ9-THC was co-administered as CBD.

During the escalation phase of Stage II (from days 1 to 6 to 9 [Fig 5]) all patients administered one, two and three escalated doses every 4–8 hours unless asleep. Total daily median (IQR) recorded doses of Δ9-THC of 1.5 (1, 2) mg (on Day 1) to 3 (2, 4) mg (on Day 6) to 3 (2, 4) mg (on Day 9), with the cannabis-based medicine generally being well tolerated. The combination MDCNB-01 medication dose schedule included 2.5 mg of CBD per dose (2 actuations of the pump). A step-down dose-approach per individual patient was adopted as directed by the treating clinician’s discretion. During the second treatment phase of Stage II (days 13 to 15) patients administered a median 2 to 2.5 mg of Δ9-THC every 4–8 hours unless asleep and again this was generally reported as well tolerated. As per the protocol further administration of the test cannabis-based medicine was discontinued from day 16. However, 15 patients (60%) received compassionate use of the MDCNB-01 on day 16 for an additional 24 hours.

The median daily amounts of Δ9-THC of MDCNB-01 administered from day 1 to day 15 are presented in Fig 5. All participants were instructed to document all doses of analgesics and the cannabis-based medicine administered in a provided medication diary. Furthermore, adherence was assessed by the clinical nursing team. The plasma concentration of cannabinoids was determined only on days 1, 4, 7, 10, 13, 16 and 30.

### Numeric pain rating scale scores

**Fig 6:**
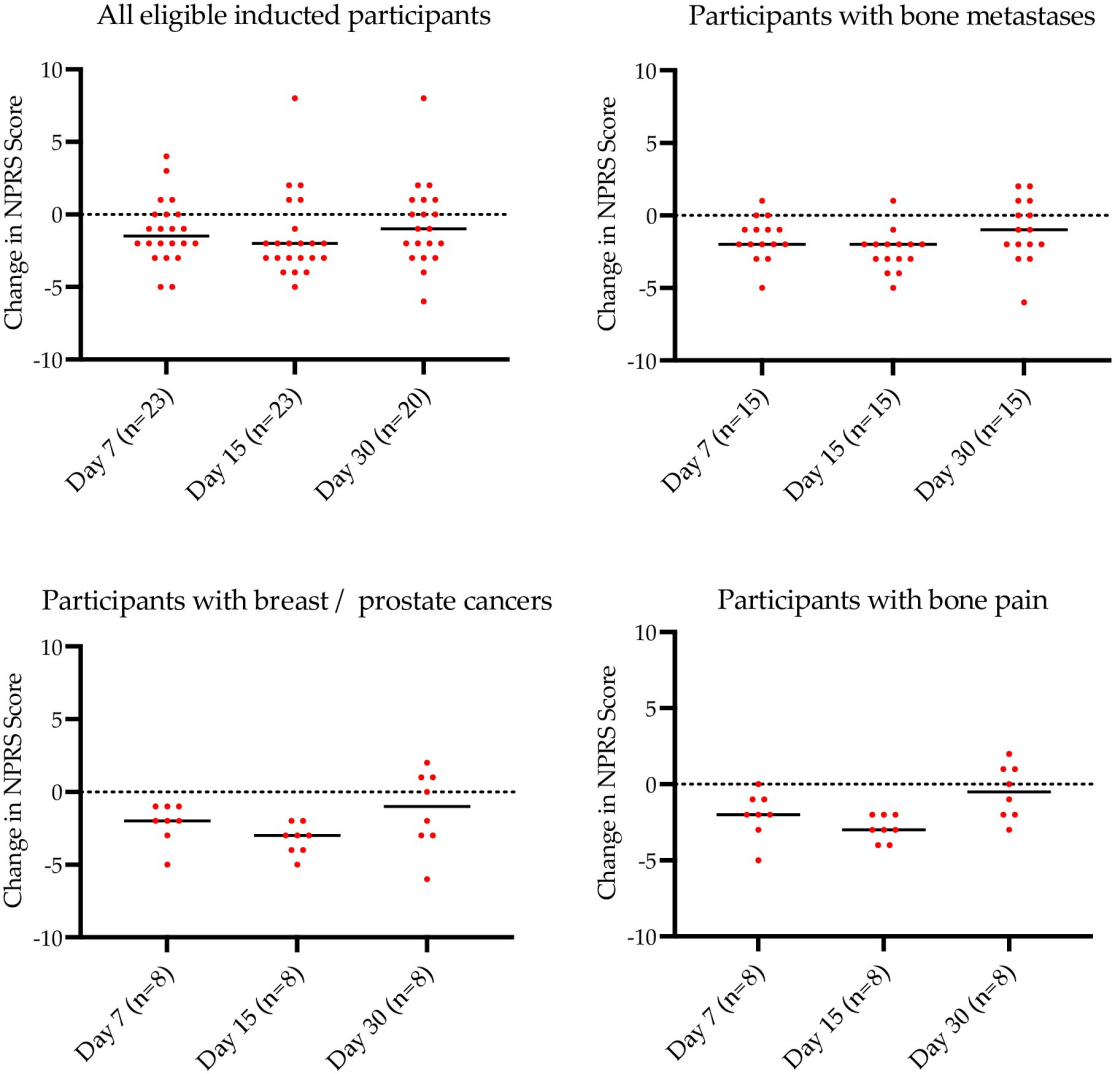
Median change from baseline NPRS scores for all patients, those with bone metastases, those with breast/prostate cancer and those with bone pain. Dots represent individual patient data; the line is median change.

There was a reduction in pain overall for the study cohort of 12% by the end of the treatment phase (Fig 6). In the sub-group of patients with breast and prostate cancers with bone metastasis the median score from baseline to 15 days was 7.5 to 3.5, an unadjusted pain improvement from baseline of approximately 40%. The improvement (re rescue medications) in pain from baseline to day 15 was 33%. From the end of the treatment phase (day 15) following discontinuation of MDCNB-01 to the end of the monitoring phase on day 30, there was a change in mean pain scores which corresponded to an overall worsening of pain scores for all patients of approximately 13% from baseline values (Fig 6).

## Discussion

This study demonstrated that the administration of the investigative cannabis-based medicine (MDCNB-01) was generally safe and tolerated in a short-term exposure in a cohort of patients with advanced incurable cancers with controlled pain or intractable pain despite opioid treatment.

Moreover, results from patient-reported outcome questionnaires suggested that patient functioning improved clinically for emotional functioning, fatigue, dyspnea, insomnia, and appetite loss, especially in those patients with improved pain management with the cannabis-based medicine, above what the maximal pain management was already providing.

The water-soluble nanoparticle cannabis-based medicine formulation demonstrated acceptable pharmacokinetic behavior for cannabinoids consistent with safety and tolerability and preliminary analgesic benefit. There was a peak plasma concentration of less than an hour and adequate concentration to support multiple dosing every four-eight hours. An oromucosal mouth spray using 50% ethanol (and propylene glycol) does not seem to be as effective as the nanoparticle water-soluble spray. For example, comparing the PK data with that reported for the ethanol-based Δ9-THC/CBD mouth spray, MDCNB-01 achieved the same C_max_ and AUC_(0-t)_ with half the Δ9-THC/CBD administered dose. The ethanol-based mouth spray (5.4 mg Δ9-THC/5.4 mg CBD) reported C_max_ and AUC_(0-t)_ of 1.48/0.39 ng/mL and 2.99/0.82 ng/mL.h, respectively [21]. With half that dose from MDCNB-01 (2.5 mg Δ9-THC/2.5 mg CBD), we found C_max_ and AUC_(0-t)_ of 1.31/058 ng/mL and 1.71/0.65 ng/mL.h, respectively. Aviram and colleagues in their study [24] report that short term administration of CBD-dominant products could also be a useful treatment for cancer related symptoms such as pain intensity and affective and sensory pain. This notion is very much in-line with our previous exploratory pilot PK study [17] that administered an anti-inflammatory CBD-dominant nanoparticle formulation to healthy participants.

In the MDCNB-01 treatment phase, patients required an average of 6.4 mg/day THC for an effective dose, 2.7-fold lower than the 17.3 mg/day of Δ9-THC reported for an ethanol based (Δ9-THC/CBD) formulation, which achieved a similar improvement in NPRS score in an advanced cancer population [25]. The clinically effective dosage supported the PK data, regarding superior absorption with the water-soluble nanoparticle formulation.

For oro-buccal sprays there is a risk that with high volumes administered that some of the volume will be swallowed prior to absorption, especially when the dose is high. Yet, the nanoparticle water-soluble delivery technology provided one peak consistent with mostly mucosal delivery, whereas a 50% ethanol mouth spray provided two peaks and inconsistent plasma levels [21], indicating inefficient mucosal absorption and swallowing of the medicine and less effective gastrointestinal absorption.

There is significant interest in the use of cannabis for the management of chronic cancer or non-cancer pain [26,27] irrespective of any adverse outcomes that have been reported. As yet, there is minimal evidence on the prevalence or predictors for adverse events in people administered cannabis [28]. In recreational users of cannabis, coughing fits, anxiety and paranoia were the most common adverse reactions [28]. In the clinical trial setting, oral, gastrointestinal, or sublingual administered cannabis, was associated with nausea, fatigue, vertigo/hallucinations, diarrhea, constipation, and dry mouth [29–33]. In our study, the administration of a nanoparticle water soluble cannabis-based medicine resulted in mild drowsiness, fatigue, and nausea. Drug tolerability was established at 2–8 sprays, namely 2.5 mg—10 mg each of Δ9-THC and CBD every 4–8 hours, which reported no evidence that the cannabis formulation increased the risk of serious adverse events. An independent safety monitoring panel concluded at the end of Stage I of this study, that there were no safety issues that would impede continued development of the study’s cannabis-based medicine. The oro-buccal delivered cannabis-based medicine administered as a water-soluble nanoparticle is of significant clinical interest given that this formulation was a self-titrated medicine, that showed preliminary analgesic efficacy in a subgroup of patients.

There are clearly however, limitations to this study. The sample was small with an open label pilot design with no comparator that included only patients with advanced cancer, with intractable pain unrelieved by opioids. There was a substantial variation in eligible patient cancer diagnoses that produced a largely heterogenous study group. Furthermore, patients presented with multiple and overlapping types of pain. The missing data were not imputed, therefore our assessment of differences in quality of life may have some bias and should be interpreted with caution.

In this cohort of patients with complex advanced cancers the adverse events encountered were similar to those commonly reported from other studies that have administered a cannabis-based medicine [34]; with the most common being drowsiness, fogginess, fatigue, nausea and vomiting. The level of emetogenicity in cancer varies based on different factors and the incidence in this study was 36%. In 8 (32%) patients that developed nausea, the causal attribution was probably / possible associated with the administered cannabis-based medicine. In 6 (24%) patients vomiting was concomitantly reported with nausea, with one patient reporting persistent nausea and vomiting that interrupted the further administration of the cannabis-based medicine. Notwithstanding this, this study of single and multiple cannabis doses that were oro-buccal administered, demonstrated a relatively good overall safety and tolerance profile.

Various clinical investigations with administered cannabis-based medicines via the gastrointestinal tract have reported limited tolerability and efficacy in a variety of indications [35]. For smoking and vaping the frequency of delivery and the magnitude of exposure to a drug can also influence the abuse potential and safety profile.

Numerous animal [36–38] and human studies [25,39–41] have reported the synergistic analgesic effects of concomitant administration of opioids and cannabinoids. In the present study, patients reported an improvement in pain scores over the course of the intervention phase of the study of approximately 12% from baseline. Notwithstanding, all patients diagnosed with bone metastasis reported a reduction in pain scores at the end of the treatment phase. In participants with a diagnosis of metastatic breast and prostate cancers (only to bone) also reported a reduction in pain scores (adjusted for rescue medications) of 33% (unadjusted of 40%) during the escalation and treatment phases (p<0.01) with minimal median increases in MMeq and rescue medications as compared to the cohort overall.

Although this study was not placebo controlled, the improvement in pain was consistent with a recent systematic and meta-analysis that concluded that cannabis-based medicines could probably achieve pain relief of 30% or greater compared with placebo. Interestingly in the metastatic breast and prostate cancer group cessation of MDCNB-01 use resulted in an average increase in the pain score of 13%.

## Conclusions

This report described a single ascending dose (Stage I and multiple ascending doses (Stage II) of a water-soluble Δ9-THC/CBD nanoparticle formulation administered to advanced cancer patients with intractable pain as a co-analgesic.

There was an overall small improvement in average NPRS pain scores over the study treatment period from baseline. The significant improvement in average adjusted NPRS pain scores of 33% above that provided by standard analgesics was recorded for an eligible subgroup of participants with a diagnosis of metastatic breast and prostate cancers (only to bone). We acknowledge that in this preliminary study the secondary endpoint of analgesic clinical efficacy by the cannabis-based medicine will require further confirmatory data from a robust study, albeit the positive signal that was observed.

## Supporting information

S1 Table Participant baseline demographic and clinical characteristics diagnosed with advanced incurable malignancy with controlled pain for Stage I (n = 5 [100%]). (DOCX)

S2 Table Participant baseline demographic and clinical characteristics diagnosed with advanced incurable malignancy with uncontrolled pain for Stage II (n = 25 [100%]). (DOCX)

S3 Table Participant baseline clinical characteristics for Stage II (n = 25). (DOCX)

S4 Table Summary of Stage I two dose-approach of n = 5 (100%) (one dose escalating to three doses) cannabinoid pharmacokinetic parameters. (DOCX)

S5 Table Treatment emergent adverse events affecting one or more participants during the MAD Stage II of the pilot trial (n = 25). (DOCX)

S1 File Tasks and procedures. (DOCX)

S2 File Phase I trial protocol v6_5.10.17. (PDF)

S3 File Vitetta_trendstatement_TREND_Checklist (1). (PDF)
