# Supplementary material for: Pilot clinical and pharmacokinetic study of Δ9-Tetrahydrocannabinol (THC)/Cannabidiol (CBD) nanoparticle oro-buccal spray in patients with advanced cancer experiencing uncontrolled pain
Source: PLoS One. 2022 Oct 14;17(10):e0270543. doi: 10.1371/journal.pone.0270543 (PMC9565400; doi:10.1371/journal.pone.0270543)
Supplement: S1 Table — (DOCX) [file pone.0270543.s001.docx]

| **Demographics Stage I** | **Number**  **n (%)** |
| --- | --- |
| **Sex** |  |
| Males | 2 (40%) |
| Females | 3 (60%) |
| **Age** | **Median (IQR)** |
| Years old | 62.0 (56.0, 73.0) |
| **Ethnicity** | |
| European | 5 (100%) |
| **Cancer Diagnosis** | **Number (%)** |
| Glioblastoma | 1 (20%) |
| Lung | 1 (20%) |
| Myeloma | 1 (20%) |
| Thyroid | 1 (20%) |
| Prostate | 1 (20%) |
