# Supplementary material for: Pilot clinical and pharmacokinetic study of Δ9-Tetrahydrocannabinol (THC)/Cannabidiol (CBD) nanoparticle oro-buccal spray in patients with advanced cancer experiencing uncontrolled pain
Source: PLoS One. 2022 Oct 14;17(10):e0270543. doi: 10.1371/journal.pone.0270543 (PMC9565400; doi:10.1371/journal.pone.0270543)
Supplement: S2 Table — (DOCX) [file pone.0270543.s002.docx]

| **Demographics Stage II** | **Number**  **n (%)** |
| --- | --- |
| **Sex** |  |
| Males  Females | 10 (40%)  15 (60%) |
| **Age** | **Mean (SD)** |
| Years old | 55.9 (11.9) |
| **Ethnicity** | **Number (%)** |
| European  Hispanic/Latino  East Asian | 20 (80%)  1 (4%)  4 (16%) |
| **Cancer Diagnosis** | **Number (%)** |
| Breast | 7 (28%) |
| Non-Small Cell Lung Cancer (NSCLC)  Lung | 4 (16%) |
| Gastrointestinal | 4(16%) |
| Hematological | 3 (12%) |
| Pancreas | 2(8%) |
| Ovaries | 2(8%) |
| Melanoma | 1(4%) |
| Central Nervous System | 1(4%) |
| Prostate | 1(4%) |
