# Supplementary material for: Pilot clinical and pharmacokinetic study of Δ9-Tetrahydrocannabinol (THC)/Cannabidiol (CBD) nanoparticle oro-buccal spray in patients with advanced cancer experiencing uncontrolled pain
Source: PLoS One. 2022 Oct 14;17(10):e0270543. doi: 10.1371/journal.pone.0270543 (PMC9565400; doi:10.1371/journal.pone.0270543)
Supplement: S3 Table — (DOCX) [file pone.0270543.s003.docx]

| **Type of Primary Cancer*** | **Number n (%)** | **Type of pain****  **(n)** |
| --- | --- | --- |
| Breast + bone metastasis | 5 (20%) | Neuropathic (2)  Neuropathic, Nociceptive (1)  Bone pain (2) |
| Breast + lung + bone metastasis  Breast + liver + bone metastasis | 1 (4%)  1 (4%) | Neuropathic, Bone pain  Neuropathic, Bone pain |
| NSCLC + brain + bone metastasis  NSCLC + bone metastasis  NSCLC + adrenal gland + liver metastasis  Lung + bone metastasis | 4 (16%) | Neuropathic, Visceral, Neuropathic, Bone pain  Neuropathic, Nociceptive, Somatic, Bone pain  Neuropathic, Nociceptive, Somatic, Bone pain  Nociceptive, Visceral, Bone pain |
| Gastrointestinal   - Oropharyngeal + lung metastasis + lymph nodes - Oesophageal + liver + lung metastasis - Large bowel + peritoneum + lung + bone metastasis - Appendix mucinous + peritoneum metastasis | 4 (16%) | Neuropathic  Nociceptive, Visceral, Neuropathic  Neuropathic, Nociceptive, Visceral  Neuropathic |
| Hematological   - Diffuse large cell lymphoma - CLL + SCC scalp metastasis - Multiple Myeloma + BPH | 3 (12%) | Neuropathic  Neuropathic  Chronic Bone pain |
| Pancreas + lung + liver + adrenal gland  Pancreas + left adrenal + peritoneum + mediastinum + para-aortic and mesenteric lymph node metastasis | 1 (4%)  1 (4%) | Nociceptive, Visceral, Neuropathic  Neuropathic, Visceral |
| Ovaries + liver + lung metastasis  Ovaries + breast + groin + abdominal lymph nodes metastasis | 1 (4%)  1 (4%) | Nociceptive, Visceral, Bone pain  Nociceptive, Visceral |
| Melanoma + bone metastasis | 1 (4%) | Neuropathic |
| Central Nervous System | 1 (4%) | Neuropathic |
| Prostate + bone metastasis | 1 (4%) | Neuropathic |

*All eligible enrolled patients were diagnosed with advanced incurable malignancy

with intractable pain unrelieved by opioids.

**Some patients presented with more than one type of pain.

NSCLC = Non-Small Cell Lung Cancer; CLL = Chronic Lymphocytic Leukemia;

SCC = Squamous Cell Carcinoma; BPH = Benign Prostatic Hypertrophy;
