# Supplementary material for: Pilot clinical and pharmacokinetic study of Δ9-Tetrahydrocannabinol (THC)/Cannabidiol (CBD) nanoparticle oro-buccal spray in patients with advanced cancer experiencing uncontrolled pain
Source: PLoS One. 2022 Oct 14;17(10):e0270543. doi: 10.1371/journal.pone.0270543 (PMC9565400; doi:10.1371/journal.pone.0270543)
Supplement: S4 Table — (DOCX) [file pone.0270543.s004.docx]

| **Parameter** | **Day 1 (n=5)**  **2.5 mg Δ9-THC+ 2.5 mg CBD**  **Median (Min, Max)** | **Day 2 (n=5)**  **7.5 mg Δ9-THC+ 7.5 mg CBD**  **Median (Min, Max)** |
| --- | --- | --- |
| **Δ9THC^⊥^** | | |
| AUC_(0-t_) ng mL.h^−1^ | 1.71 (1.11, 6.61)* | 8.26 (2.67, 11.72) |
| C_max_ ng mL^−1^ | 1.31 (0.76, 2.94)* | 2.35 (1.09, 3.19) |
| t_max_ hours | 0.75 (0.5, 1.5)* | 1.00 (0.5, 2.0) |
| t_1/2_ hours | 0.94 (0.75, 1.14)* | 1.39 (1.30, 2.88) |
| **CBD** | | |
| AUC_(0-t)_ ng mL.h^−1^ | 0.65 (0.49, 4.06)* | 5.96 (1.51, 12.15)* |
| C_max_ ng mL^−1^ | 0.58 (0.48, 2.45)* | 1.55 (0.62, 2.25) |
| t_max_ hours | 0.75 (0.5, 1.5)* | 1.00 (0.5, 2.0) |
| t_1/2_ hours | 0.72 (0.57, 0.86)* | 1.53 (1.16, 7.06) |
| **11-OH-THC** | | |
| AUC_(0-t)_ ng mL.h^−1^ | 3.10 (2.17, 49.37) | 17.2 (7.91, 99.13) |
| C_max_ ng mL^−1^ | 2.06 (0.29, 13.8) | 3.74 (1.06, 20.4) |
| t_max_ hours | 1.00 (0.5, 1.5) | 1.50 (0.5, 2.0) |
| t_1/2_ hours | 4.05 (1.19, 5.23) | 5.31 (1.60, 8.02) |
| **COOH-THC** | | |
| AUC_(0-t)_ ng mL.h^−1^ | 126.32 (34.29, 251.29)* | 223.39 (162.57, 1172.98) |
| C_max_ ng mL^−1^ | 13.70 (6.62, 25.40)* | 26.80 (13.1, 96.0) |
| t_max_ hours | 1.25 (0.5, 2.0)* | 2.5 (1.5, 3.0) |
| t_1/2_ hours | 10.94 (2.34, 12.33)* | 10.09 (7.41, 19.23) |

^⊥^ Δ9THC = delta-9-tetrahydrocannabinol; CBD = Cannabidiol;

11-OH-THC = 11-hydroxy-tetrahydrocannabinol;

COOH-THC = carboxy-tetrahydrocannabinol

AUC = area under the plasma concentration versus time curve, from time zero to the last measurable concentration at t = 6 hr;
Cmax = maximum measured plasma concentration over the time span specified;
Tmax = time of maximum measured plasma concentration;
t1/2 = time required for the concentration of the drug to halve;
* for n=4 patients only due to one sample not evaluable.
