# Supplementary material for: Pilot clinical and pharmacokinetic study of Δ9-Tetrahydrocannabinol (THC)/Cannabidiol (CBD) nanoparticle oro-buccal spray in patients with advanced cancer experiencing uncontrolled pain
Source: PLoS One. 2022 Oct 14;17(10):e0270543. doi: 10.1371/journal.pone.0270543 (PMC9565400; doi:10.1371/journal.pone.0270543)
Supplement: S5 Table — (DOCX) [file pone.0270543.s005.docx]

| **Adverse Event Description** | **Mild**  **n (%)** | **Moderate**  **n (%)** | **Severe**  **n (%)** |
| --- | --- | --- | --- |
| Auditory hallucination | 1 (4%) | - | - |
| Burning throat | 1 (4%) | - | - |
| Constipation | 2 (8%) | 1 (4%) | - |
| Dizziness | 10 (40%) | 1 (4%) | - |
| Drowsiness | 17 (68%) | 11 (44%) | 4 (16%) |
| Dry mouth | 1 (4%) | 3 (12%) | 2 (8%) |
| Fatigue | 1 (4%) | 5 (20%) | 3 (12%) |
| Fogginess | 5 (20%) | 2 (8%) | 1(4%) |
| Hallucinations | - | 2 (8%) | - |
| Impaired Concentration | 1 (4%) | - | - |
| Lethargy | - | 1 (4%) | 1 (4%) |
| Nausea | 9* (36%) | 5 (20%) | 1 (4%) |
| Nightmare | 1 (4%) | - | - |
| Numbness bottom lip | 1 (4%) | - |  |
| Pain crisis post coming off IP* | - | - | 1 (4%) |
| Restless at night | - | 1 (4%) | - |
| Vivid dreams | 1 (4%) | - | - |
| Vomiting | 5 (20%) | 1 (4%) | 3 (12%) |

*One patient developed nausea after administering one dose of the cannabis-based

medicine (2 sprays) as a standardized AE [20].
