# Supplementary material for: Pilot clinical and pharmacokinetic study of Δ9-Tetrahydrocannabinol (THC)/Cannabidiol (CBD) nanoparticle oro-buccal spray in patients with advanced cancer experiencing uncontrolled pain
Source: PLoS One. 2022 Oct 14;17(10):e0270543. doi: 10.1371/journal.pone.0270543 (PMC9565400; doi:10.1371/journal.pone.0270543)
Supplement: S1 File — (DOCX) [file pone.0270543.s006.docx]

|  | | | **Stage 2 Dose Escalation Phases (Period)** | | | | | | | | | | | **Stage 2 Treatment Phase (Period)** | | | | | | | **Follow Up Post**  **Treatment Phase** | | | |
| --- | --- | --- | --- | --- | --- | --- | --- | --- | --- | --- | --- | --- | --- | --- | --- | --- | --- | --- | --- | --- | --- | --- | --- | --- |
|  |  |  | **Phase-1**  **1 Dose** | | | **Phase-2**  **2 Doses** | | | | **Phase-3**  **3 Doses** | | | | **Established Dose** | | | | | | |  |  |  |  |
| **Site** | **TASK \ DAYS** | **−2–0** | **1** | **2** | **3** | | **4** | **5** | **6** | | **7** | **8** | **9** | | **10** | **11** | **12** | **13** | **14** | **15** | | **16** |  | **30** |
| RNSH * | Screening | 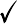 |  |  |  | |  |  |  | |  |  |  | |  |  |  |  |  |  | |  |  |  |
| RNSH | Informed Consent | 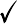 |  |  |  | |  |  |  | |  |  |  | |  |  |  |  |  |  | |  |  |  |
| RNSH | Inclusion/Exclusion Criteria | 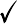 |  |  |  | |  |  |  | |  |  |  | |  |  |  |  |  |  | |  |  |  |
| RNSH | Urine sample collection | 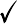 |  |  |  | |  |  |  | |  |  |  | |  |  |  |  |  |  | |  |  |  |
| RNSH | Medical history | 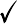 |  |  |  | |  |  |  | |  |  |  | |  |  |  |  |  |  | |  |  |  |
| RNSH | Concomitant medications | 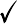 | 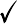 | 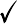 | 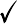 | | 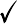 | 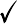 | 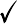 | | 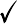 | 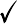 | 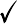 | | 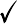 | 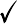 | 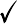 | 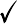 | 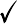 | 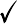 | | 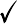 |  | 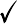 |
| HOME/RNSH | NPRS score * | 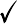 |  | 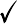 | 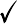 | | 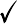 | 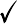 | 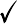 | | 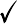 | 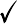 | 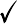 | | 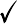 | 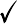 | 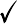 | 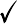 | 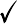 | 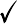 | | 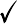 |  | 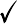 |
| HOME/RNSH | EORTC QLQ-30 * | 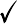 |  |  |  | |  |  |  | | 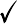 |  |  | |  |  |  |  |  | 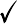 | |  |  | 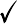 |
| HOME/RNSH | Medication diary completion |  | 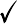 | 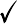 | 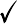 | | 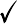 | 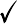 | 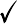 | | 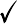 | 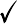 | 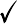 | | 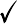 | 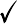 | 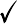 | 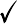 | 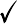 | 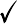 | | 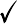 |  |  |
| RNSH | Drug dispensing |  | 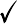 |  |  | | 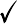 |  |  | | 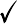 |  |  | | 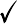 |  |  | 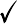 |  |  | |  |  |  |
| RNSH | Outpatient visits |  | 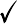 |  |  | | 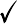 |  |  | | 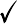 |  |  | | 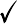 |  |  | 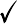 |  |  | | 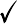 |  | 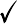 |
| RNSH | MMeq recording (study staff) * |  | 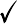 |  |  | | 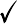 |  |  | | 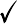 |  |  | | 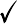 |  |  | 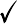 |  |  | | 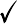 |  | 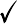 |
| RNSH | Blood sample collection |  | 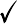 |  |  | | 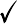 |  |  | | 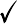 |  |  | | 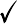 |  |  | 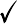 |  |  | | 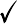 |  | 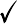 |
| HOME/RNSH | AEs monitoring/recording | 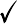 | 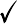 | 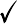 | 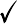 | | 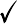 | 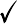 | 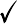 | | 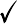 | 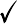 | 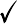 | | 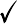 | 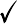 | 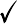 | 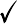 |  |  | |  |  |  |
| HOME/RNSH | SAEs monitoring/recording |  |  |  |  | |  |  |  | |  |  |  | |  |  |  |  |  |  | |  |  |  |

* RNSH = Royal North Shore Hospital; NPRS = Numerical Pain Rating Scale;

EORTC-QLQ-30 = EORTC core quality of life questionnaire QLQ-30(v3); MMeq = Morphine Milligram equivalents;
