# Supplementary material for: Pilot clinical and pharmacokinetic study of Δ9-Tetrahydrocannabinol (THC)/Cannabidiol (CBD) nanoparticle oro-buccal spray in patients with advanced cancer experiencing uncontrolled pain
Source: PLoS One. 2022 Oct 14;17(10):e0270543. doi: 10.1371/journal.pone.0270543 (PMC9565400; doi:10.1371/journal.pone.0270543)
Supplement: S2 File — (PDF) [file pone.0270543.s007.pdf]

The buccal administration of a *Cannabis sativa L./Cannabis indica L.* extract as an adjunct to opioid analgesia for the management of intractable pain in patients diagnosed with advanced cancer: a safety, tolerability and exploratory end-point pilot study

INVESTIGATORS

Principal Investigator (PI): Professor Stephen Clarke. Medical Oncologist; Northern Sydney Cancer Centre.

Associate Investigator 1 (AI1): Professor Paul Glare. Director Pain Management Research Institute Michael J Cousins Pain Management and Research Centre, Douglas Building, Royal North Shore Hospital.

Associate Investigator 2 (AI2): A/Professor Charles Brooker. Michael J Cousins Pain Management and Research Centre, Douglas Building, Royal North Shore Hospital, Reserve Rd. St Leonards, NSW, 2065.

Associate Investigator 3 (AI3): Professor Andrew McLachlan. Program Director of the NHMRC Centre for Research Excellence in Medicines and Ageing; The University of Sydney. A15 – Pharmacy and Bank Building, The University of Sydney, NSW, 2006

Associate Investigator 4 (AI4): Dr Sean Hall. CEO/MD; Medlab Clinical Ltd.

Associate Investigator 5 (AI5): Professor Luis Vitetta is Director of Medical Research at Medlab Clinical Ltd. and The University of Sydney, Sydney Medical School.

Research Associate 1: Sally McCowatt. Clinical Research Coordinator;
Northern Cancer Centre, Level 1, Royal North Shore Hospital.

Research Associate 2: Linda Critchley. Coordinator of Clinical Research; Pain Management Research Institute. Pain Management and Research Centre, Douglas Building, Royal North Shore Hospital.

Independent Statistician: **Dr Belinda Butcher** BSc(Hons) MBiostat PhD CMPP. Director - Biostatistics and Medical Writing | WriteSource Medical Pty Ltd

STATEMENT OF COMPLIANCE

This document is a protocol for a clinical research study. The study will be conducted in compliance with all stipulations of this protocol, the conditions of ethics committee approval, the NHMRC National Statement on Ethical Conduct in Human Research (2007) and the Note for Guidance on Good Clinical Practice (CPMP/ICH-135/95).

Contents

STATEMENT OF COMPLIANCE 1

PROTOCOL SYNOPSIS 4

GLOSSARY OF ABBREVIATIONS........ 9

1. STUDY MANAGEMENT 11

2. INTRODUCTION AND BACKGROUND 13

2.2 RESEARCH QUESTION 19

2.3 RATIONALE FOR CURRENT STUDY 19

3 STUDY OBJECTIVES 19

4. STUDY DESIGN 19

5. STUDY TREATMENTS 25

5.1 TREATMENT ARM 25

5.1.1 DESCRIPTION 25

5.1.2 DOSAGE AND ROUTE OF ADMINISTRATION 25

5.2 PREPARATION AND ADMINISTRATION OF STUDY DRUG 26

5.2.1 PREPARATION 26

5.2.2 ADMINISTATION 26

5.3 MEASUREMENT OF PARTICIPANT COMPLIANCE 27

5.4 EXCLUDED MEDICATIONS AND TREATMENTS 27

6. PARTICIPANT ENROLMENT 27

6.1 RECRUITMENT 27

6.2 ELIGIBILITY CRITERIA 28

6.2.1 INCLUSION CRITERIA 28

6.2.2 EXCLUSION CRITERIA 26

6.3 INFORMED CONSENT PROCESS 29

7. STUDY VISITS AND PROCEDURES SCHEDULE 32

8. CLINICAL AND LABORATORY ASSESSMENTS 36

9. ADVERSE EVENT REPORTING 36

9.1 DEFINITIONs 36

9.3 ELICITING ADVERSE EVENT INFORMATION 37

9.4 SERIOUS ADVERSE EVENT REPORTING 37

9.4.1 SEA's 37

9.4.2 SUSARs 37

9.5 SPECIFIC SAFETY CONSIDERATIONS 37

10. STATISTICAL METHODS 38

10.1 SAMPLE SIZE ESTIMATION 38

10.2 POPULATION TO BE ANALYSED 38

10.3 STATISTICAL ANALYSIS PLAN 38

10.4 INTWERIM ANALYSES 38

11. DATA MANAGEMENT 38

12. ADMINISTRATIVE ASPECTS 39

13. USE OF DATA AND PUBLICATIONS POLICY 40

14. REFERENCES 38

15. APPENDICES 42

PROTOCOL SYNOPSIS

Title	The buccal administration of a *Cannabis sativa* L./*Cannabis indica L.* extract as an adjunct to opioid analgesia for the management of intractable pain in patients diagnosed with advanced cancer: a safety, tolerability and exploratory end-point pilot study.
Objectives	**Primary:**<br>To investigate the safety, tolerability and pharmacokinetics (PK) of a cannabis (THC:CBD) extract by buccal administration at two dose levels in people with cancer pain.<br><br>**Secondary:**<br>Exploratory pilot study to evaluate the analgesic effects of a THC:CBD extract as an adjunct to opioid analgesia in the management of pain in patients with moderate to severe cancer-related pain measured by:<br>— Pain Numerical Rating Scale (NRS).<br><br>— Rescue analgesia (opioid) requirement.<br><br>**NOTE:**<br>1. Primary end-point to be clinician assessed for safety and tolerability, and pharmacokinetic parameters (related to systemic exposure of THC and CBD).<br>2. Secondary end-points to be clinician and patient self-reported.<br>3. The requisite will also be for careful tracking of subject opioid analgesic use. This will be best calculated as morphine equivalent (MMeq) doses and record how changes occur with the administration of the THC+CBD extract.<br>4. If an unforeseen adverse event is reported, the PI may require the participant to remain admitted for extended monitoring.

Study achievement(s)	A safe and tolerable outcome for this study is to progress the acceptance of a standardised cannabis extract for the management of intractable pain in cancer patients.
Study Design	The study has 2 stages. The study design is fully explained in section 4.2, page 17-22 and charted on pages 30 - 32 of this protocol document.<br><br>**STAGE 1 (n= 5 people with advanced cancer):**<br>Day -2:<br>• participant's assessments and on satisfying inclusion and exclusion criteria and completion of signed informed consent. Baseline blood sample collection.<br>Day 1:<br>• Open label single treatment group pharmacokinetic arm of the study administering standard a dose of 2.6 mg THC and 2.4 mg CBD in 0.3 mL (representing 1 "standard dose" of 2 actuations from the buccal spray delivery system).<br>Day 2 (consecutive):<br>• Administering 7.8 mg THC and 7.2 mg CBD in 0.9 mL (representing 3 "standard doses" of 6 actuations from the buccal spray delivery system).<br>Day 1 and 2: Participants remain under clinical supervision in hospital for 48 hours [2 nights] to carefully assess safety and tolerability of the product.<br>If an unforeseen adverse event is reported, the PI may require the participant to remain admitted for extended monitoring.<br>NOTE: Blood collections for safety and pharmacokinetic sampling for the 2 consecutive days detailed in Section 7 Page 30 Table 2 of this protocol.<br><br>**DSMB to review safety and tolerability data before continuation to the next stage.**<br><br>**STAGE 2 (n=25 participants with advanced cancer and intractable pain not responsive to opioid analgesic medicines):**<br>Consists of (i) dose escalation phase; (ii) dose treatment phase;<br>(iii) follow-up phase.<br>Please note: If an unforeseen adverse event is reported, the PI may require the participant to remain admitted for extended monitoring.<br>(i) Dose escalation phase:<br>• Day -2: Participants assessments and on satisfying inclusion and exclusion criteria and completion of signed informed consent. Baseline blood sample collection. Participants will be advised that they will be hospitalised for days 1, 4 and 7 of this stage of the study.

	• On Days 1 to 3: Dispense new cannabis vial. Administer 2 actuations of the pump (delivering 2.6 mg THC+2.4 mg CBD in 0.3 mL) every 4 hours (unless asleep) repeat for 3 days. Participants to complete questionnaires at the end of each day in the case report form. On day 1 of stage 2 participants will be hospitalized for 1 night at RNSH and assessed. At the discretion of the PI (based on tolerability) participants may require longer observational periods in hospital.<br>• Return to RNSH on day 4 for admission to RNSH and for assessments. Return used cannabis medicine vial. Dispense new cannabis vial. Increase dose to two doses (4 actuations of the pump delivering 5.2 mg THC+4.8 mg CBD in 0.6 mL) every 4 hours repeat for 3 days. Blood sample collection. On day 4 of stage 2 participants will be hospitalized for 1 night at RNSH. At the discretion of the PI participants may require longer observational periods in hospital.<br>• Return to RNSH on day 7 for admission to RNSH and for assessments. Return used cannabis medicine vial. Dispense new cannabis vial. Increase dose to three doses (6 actuations of the pump delivering 7.8 mg THC+7.2 mg CBD in 0.9 mL) every 4 hours repeat for 3 days. Blood sample collection.<br>(ii) Treatment phase (ongoing):<br>• Return to RNSH on day 10 for assessments. Return used cannabis medicine vial. Participants undergo physical examination. Participants return completed Phase 2 Case Report Form (CRF). Dispense new cannabis vial. Administer, either, one, two or three doses for 3 days once every 4 hours as indicated by clinician. Blood sample collection.<br>• Return to RNSH on day 13 for assessments. Return used cannabis medicine vial. Dispense new cannabis vial. Continue administration of cannabis medicine at the same dose as directed by clinician. Blood sample collection. Final dose of investigative product will occur at the end of day 15.<br>• Return to RNSH on day 16 for assessments. Return used cannabis medicine vial. Participants undergo physical examination. Participants return completed Phase 2 Case Report Form (CRF). Blood sample collection.<br><br>(iii) Follow-up phase:<br><br>• Return to RNSH on Day 30. Participants will be followed-up after completing dose escalation and treatment phases 2-weeks post completion. Participants complete questionnaires and a blood sample will be collected.<br><br>**DSMB second meeting to review all data before completing report.**
Planned Sample Size	30

Selection Criteria	STAGE 1:<br>People living with advanced cancer<br><br>STAGE 2:<br>Adult male or female participants who are receiving strong opioid analgesics (i.e., morphine, hydromorphone, oxycodone) for at least one week to relieve pain associated with incurable malignancy and who have given written informed consent will be screened for study entry.<br>The prescribed Morphine Milligram equivalents (MMEq) / day dose should be less than 60 mg MMeq/day.<br>Eligible participants for the Stage 2 of the study will record at least 1 pain severity score of 4 or above on a 0-10 Numerical Rating Scale (NRS) on the -2 day assessment day (day -2).
Study Procedures	For eligible participants invited to participate in **STAGE 1** safety, tolerability and pharmacokinetic (PK) part of the study.<br>**STAGE 1 procedures:**<br>**Day -2:**<br>• Sign informed consent<br>• Assessment of eligibility<br>• Study related assessments (e.g., record current morphine equivalent)<br>• MMeq doses, complete a study diary, recording pain score, background medication and all additional breakthrough analgesia on each day during the baseline period. Blood sample collected.<br>**Day 1:**<br>• Admission into RNSH for overnight stay for 2 nights.<br>• Commence a buccal administered prescribed standard dose of the cannabis medicine containing the THC:CBD extract as two actuations of the drug delivery pump (2.6 mg THC+2.4 mg of CBD/0.3 mL) one time only.<br>• Safety and tolerability assessments.<br>• Blood sample collections to measure plasma THC and CBD and 11-hydroxy-THC and 11-nor-9-carboxy-COOH-THC concentrations at the following time points: (baseline) 0, 30, 60, 90, 120, 150, 180, 240, 360 minutes, 12 hours, 24 hours.<br>• <u>Note:</u> These sampling times align with the expected PK profile of cannabinoids of interest after Sativex buccal delivery (seen in the paper by Karschner *et al*. Clin Chem 2011).<br><br>**Day 2 (consecutive):**<br>• Participants commence buccal administered 3 prescribed doses (6 actuations of the pump delivering 7.8 mg THC+7.2 mg CBD/0.9 mL) of the cannabis product. First dose at 0 minutes, second dose at 2 minutes and third dose at 4 minutes.<br>• Safety and tolerability assessments.<br>• Blood sample collections to measure plasma THC and CBD and 11-hydroxy-

	THC and 11-nor-9-carboxy-THC concentrations at the following time points: (baseline) 0, 30, 60, 90, 120, 150, 180, 240, 360 minutes, 12 hours, 24 hours.<br>• <u>Note:</u> that the PI will oversee the patient admitted for two nights of stage 1 and may keep the patient in hospital if deemed relevant beyond the mandatory admission (2 nights) based on adverse changes to the investigative drug.<br>Participants released from RNSH after 48 hours. Eligible participants who report future opioid intolerance will be invited to participate in Stage 2 of the Dose Escalation and Treatment phases of the study.<br><br>**STAGE 2 procedures:**<br>**Day -2 at RNSH:**<br>• Sign informed consent.<br>• Assessment of eligibility.<br>• Study related assessments (e.g., record current morphine equivalent (MMeq) doses, complete a study diary, recording pain score, background medication and all additional breakthrough analgesia on each day during the baseline period). Collect blood sample.<br>**Days 1-9 (Dose Escalation Phase):**<br>• On Days 1 to 3: administer one standard dose, 2 actuations of the pump (delivering 2.6 mg THC+2.4 mg CBD in 0.3 mL) every 4 hours repeat for 3 days.<br>• Return to RNSH on day 4 for study related assessments and on this day increase dose to two standard doses, 4 actuations of the pump (delivering 5.2 mg THC + 4.8 mg CBD in 0.6 mL) every 4 hours repeat for 3 days. Return used cannabis medicine vial. Dispense new cannabis vial.<br>• Return to RNSH on day 7 for study related assessments and on this day increase dose to three standard doses (6 actuations of the pump delivering 7.8 mg THC+7.2 mg CBD in 0.9 mL) every 4 hours repeat for 3 days. Return used cannabis medicine vial. Dispense new cannabis vial.<br>• Collect blood samples on days 1, 4, 7.<br>• <u>Note:</u> that the PI will oversee the patient admitted to RNSH on Days 1, 4 and 7 for one night of the dose escalation phase of Stage 2 and may keep the patient in hospital for a longer period of time if deemed relevant beyond the mandatory admission (1 night) based on adverse changes to the investigative drug. See Table 3 on page 32.<br>**Days 10 – 15 (Treatment Phase):**<br>• Return to RNSH on day 10 for study related assessments. Return used cannabis medicine vial. Dispense new cannabis vial. Administer, either one, two or three doses once every 4 hours repeat for the next 2 days as directed by PI. Blood collection.<br>• On day 13 return to RNSH for study related assessments and return used cannabis medicine vial. Dispense new cannabis vial and begin administration on this day one, two or three doses every 4 hours repeat for the next 2 days at the same previous dose as directed by PI. Collect blood sample.

	**Days 16 – 30 (Follow-up Phase):**<br>• On day 16 return to RNSH for study related assessments and return used cannabis medicine vial. Return completed CRF. Blood collection.<br>• Participants will be followed-up after completing dose escalation and treatment phases at day 30 (2-weeks post completion) at RNSH. Complete study related assessments.<br>• Blood collection.<br>• Please note: The last treatment dose is at the end of day 15.
Statistical Procedures Sample Size Calculation Analysis Plan:	1. PK studies of new drugs, involves taking several blood samples over a period of time from study participants to determine how the body handles the substance. These studies provide critical information about new drug. As such the current protocol is an exploratory end-point pilot study with limited inferential statistical tests proposed within this protocol. To investigate the pharmacokinetics and systemic exposure of an investigational drug in a pilot exploratory study for a single dose versus 3 doses typically in PK studies the number of healthy participants or with cancer can be 4 to 10 [Clin Ther 2016;38(9):2106-15; Cancer Sci 2014;105(3):347-53] (1,2). We have chosen for the PK study a sample of n = 5.<br>2. Sample size calculation for the dose escalation–treatment phase of the study was assigned a power of 80% for a moderate effect size of 0.5 and a significance of 0.05 (one tail) and this gave a sample size of n=25.<br>3. The dose escalation–treatment (stage 2) of the study: for the primary outcome of safety and tolerability the assessments will be descriptive. For the two secondary efficacy variables (i.e., NRS pain score and use of breakthrough medication), the Hochberg (3) method will be used to test the global hypothesis for an exploratory treatment effect on pain. The pre-dose NRS score will be the average of the 3 previous NRS scores in any one week as per opioid use as indicated in the inclusion criteria (see page 25 of this protocol). The pain NRS score will be the mean of the first 12 hours post-dose assessments. The change in mean NRS pain score from baseline (all days in run-in period) to the post-dosing will be analyzed using analysis of covariance (ANCOVA) (2,3). The proportion of responders (patients with >= 30% improvement from baseline to end of study NRS pain score) will be compared. A change of 3 points from an initial pain score of 4-5 is expected. The study will record a mean (SD) treatment difference from baseline with a one –sided significance of p<0.05 (5,6).<br>4. The study will also record an increase or reduction in rescue analgesia (opioid) requirements over the dose escalation– interventional phases of the study.

Duration of the study	1. Pre-screening for both Stages is 2 days prior commencement of both Stages individually.<br>2. Stage 1: 2 consecutive days of participation in hospital.<br>3. Stage 2: The treatment duration is 15 consecutive days with a follow-up appointment at day 30. The participants will be admitted overnight a minimum of 3 non-consecutive days to RNSH on days 1, 4 and 7 of the dose escalation phase.<br>4. The study (stage 1 and 2) will have an estimated duration of 9 months for completion.

GLOSSARY OF ABBREVIATIONS

ABBREVIATION	TERM
NRS	Numerical Rating Scale
QoL	Quality of Life
THC	Δ^9- tetrahydrocannabinol
CBD	Cannabidiol
PI	Principal Investigator
SD	Standard deviation
ANCOVA	Analysis of covariance

1. Study Management

1.1 Principal Investigators

Principal Investigator (PI): Professor Stephen Clarke. Medical Oncologist; Northern Cancer Institute. Level 1/38 Pacific Highway, St Leonards, NSW, 2065. Stephen.clarke@sydney.edu.au, Tel. 02 9437 1900. PI is the onsite lead investigator and will be provide prospective participants from his clinical practice and will be the specialist in charge of prescribing the cannabis extract. To this latter point, PI will have applied for NSW Health Authority to prescribe cannabis. PI will submit the research document for site authorisation.

1.2 Associate Investigators

Associate Investigator 1 (AI1): Professor Paul Glare. Director Pain Management Research Institute Michael J Cousins Pain Management and Research Centre, Douglas Building, Royal North Shore Hospital, Reserve Rd. St Leonards, NSW, 2065. Paul.glare@sydney.edu.au, Tel. 02 9463 1526.

Associate Investigator 2 (AI2): A/Professor Charles Brooker. Michael J Cousins Pain Management and Research Centre, Douglas Building, Royal North Shore Hospital, Reserve Rd. St Leonards, NSW, 2065. Charles.brooker@sydney.edu.au, 02 9463 1500. AI1 and AI2 will advise on protocol development and clinical pain management during the study. AI1 and AI2 will not have a role in management of blood samples or recording data directly nor be responsible for the safety of the pilot study. These roles will be the responsibility of the PI's research team at the oncology unit of the RNSH.

Associate Investigator 3 (AI3): Professor Andrew McLachlan. Program Director of the NHMRC Centre for Research Excellence in Medicines and Ageing; The University of Sydney. A15 – Pharmacy and Bank Building, The University of Sydney, NSW, 2006. andrew.mclachlan@sydney.edu.au, Tel. 02 9351 4452. AI3 will liaise with a RNSH pharmacy senior pharmacist who will dispense the investigational cannabis extract product to eligible participants and control all vials of the investigational product (including returns and note residual cannabis medicine volumes in the returned vials). The senior pharmacist will also be charged with maintaining a register of the cannabis vials received and dispensed and provide this information to PI.

Associate Investigator 4 (AI4): Dr Sean Hall. CEO/MD; Medlab Clinical Ltd. 66 McCauley Street, Alexandria, NSW, 2015. Sean_hall@medlab.co, Tel. 0411 603 378. AI4 will oversee and assist PI with

i) the delivery of raw cannabis material from Canada to Australia (Melbourne) and ii) the manufacture of the pharmaceutical grade micellized cannabis medicine and the transportation of the standardized fine mist spray from Melbourne to Sydney and on to the RNSH pharmacy.

Associate Investigator 5 (AI5): Professor Luis Vitetta is Director of Medical Research at Medlab Clinical Ltd. and The University of Sydney, Sydney Medical School; Medlab Clinical Ltd. 66 McCauley Street, Alexandria, NSW, 2015. Luis.vitetta@sydney.edu.au, Tel. 0402 263 316. AI5 will take overall administrative support responsibility for the study and contribute to reporting of the research project. This will occur in conjunction with the PI and AIs.

Research Associate 1: Sally McCowatt. Clinical Research Coordinator;
Northern Cancer Centre, Level 1, Royal North Shore Hospital,
St Leonards NSW 2065
Sally.McCowatt@health.nsw.gov.au
02 9463 1181

Research Associate 2: Linda Critchley. Coordinator of Clinical Research; Pain Management Research Institute. Pain Management and Research Centre, Douglas Building, Royal North Shore Hospital, Reserve Rd, St Leonards, NSW, 2065.
Linda.critchley@health.nsw.gov.au
02 9463 1533.

1.4 Statistician

Independent Statistician:
Dr Belinda Butcher BSc(Hons) MBiostat PhD CMPP
Director - Biostatistics and Medical Writing | WriteSource Medical Pty Ltd
PO Box 1521| Lane Cove NSW 2066
Email: bbutcher@writesourcemedical.com.au
Telephone: 0418 286 014

1.5 Internal Trial Committees

Please see DSMB file.

1.6 Independent Safety and Data Monitoring Committee

An independent safety and data monitoring committee will be formed (see DSMB document #13) consisting of a group of individuals with pertinent expertise in the field that will on a regular basis review all accumulated data from the ongoing clinical trial. Membership will include an independent statistician, oncologist, and research investigators with expertise in the conduct of clinical trials. The data monitoring committee will advise the sponsor regarding the:

I. Continuing safety of trial participants and those yet to be recruited to the trial.
II. Continuing validity and scientific merit of the trial.

As per NHMRC guidelines (8) the investigators/researchers will report all SAEs to the sponsor immediately (within 24 hours) in accordance with the study protocol and GCP guidelines as adopted by the TGA (9).

a) In a prompt manner communicate to all investigators information which could adversely affect the safety of participants, materially impact the continued ethical acceptability of the trial or that requires (or indicates the need for) a change to the trial protocol, including changed safety monitoring as per guidelines (9).
b) Establish safety monitoring processes that are commensurate with the risk, size, and complexity of the proposed research.
c) Be in regular communication with all other investigators.
d) Keep all investigators up to-date with safety issues in the trial in a manner that is consistent with the risk, size, and complexity of the proposed research.
e) provide to investigators the periodic information listed under section 2 to facilitate investigator submission to the relevant HREC
f) be responsible for reporting individual case safety reports (ICSR) to the TGA in accordance with expedited reporting guidelines (10).

1.7 Sponsor

Medlab Clinical Ltd (Alexandria, NSW Australia)

1.8 Funding and resources

Commercial Sponsor:
Medlab Clinical Ltd.
66 McCauley St., Alexandria, NSW. 2015
Contact Telephone: (02) 8188 0311

2. INTRODUCTION AND BACKGROUND

2.1 Background Information

The dried leaves of the *Cannabis sativa* plant have long been used for both recreational and medical purposes (11,12). *Cannabis sativa* contains a number of chemical compounds, some of which are classified as cannabinoids. Cannabinoid was the term originally used for C21 terpenophenolic compounds originally found in this plant. These compounds were found to activate cannabinoid receptors in the brain, and now this term is also used to describe other compounds that activate those receptors, even if they do not have a similar chemical structure (13). Two major types of cannabinoid receptors have been characterized: CB1 and CB2. CB1 receptors are found mainly in central and peripheral neurons, whereas CB2 receptors are found most often in immune cells. Nevertheless, CB1 receptors can be found in immune cells, whilst CB2 receptors can be found in neurons (12). These receptors, along with endogenous cannabinoid receptor agonists (endocannabinoids, molecules naturally found in and produced by the body that activate these receptors), are known collectively as the endocannabinoid system (14).

Although there are more than 60 cannabinoids in marijuana (13), 2 in particular have been the subject of most studies examining medicinal uses: delta-9-tetrahydrocannabinol (D9-THC, often referred to just as THC) and cannabidiol (CBD). THC is often called the major psychoactive component of marijuana because it appears to be responsible for the feeling of *high* reported by consumers of marijuana. In addition to its euphoriant properties, THC also has analgesic, antiemetic, anti-inflammatory and antioxidant properties (10). CBD is another major cannabinoid found naturally in the marijuana plant. Although CBD has low affinity for CB1 and CB2 receptors, at low concentrations it can antagonize CB1/CB2 agonists and may even behave as an inverse agonist (14).
Despite being labelled as *non-psychoactive* in the past, CBD has anxiolytic and antipsychotic properties. It also has anticonvulsive properties and can counteract some of the psychoactive effects of THC (9,10,12). It also has reported efficacy in the treatment of pain, although this may be due more to its anticonvulsive effects than an antinociceptive effect.

Delta-9-tetrahydrocannabinol: THC is highly lipophilic and is water insoluble. It is rapidly absorbed into the blood from inhaled marijuana smoke, with plasma levels becoming detectable within seconds and peak plasma levels noted in less than 10 minutes. Peak plasma levels are directly related to the THC content of the marijuana that is smoked (15). The bioavailability of THC from smoking marijuana varies based on depth of inhalation, puff and breath-holding duration, and is estimated to be between 10% to 35%, with higher systemic bioavailability for heavy users than occasional users.

Characterization of absorption of THC after oral administration has largely been based on studies of the pharmaceutical dronabinol, although there have been a few studies of marijuana in baked goods. Absorption after oral administration has been described as “slow and erratic,” resulting in *low and irregular* plasma levels. THC can be degraded by acid, which could potentially lower the amount available to be absorbed by the stomach. It is known to undergo extensive first-pass metabolism (16). After oral ingestion, plasma levels usually peak after 60 to 120 minutes, although in some participants it can take as long as 4 hours or more to observe peak plasma levels. Some participants can even have more than one peak after a single oral dose (16). Bioavailability after oral ingestion is approximately 6%, but with high variability between participants (17). THC can also be administered via the oral mucosa. Mean plasma levels reach the threshold of detection at 45 minutes after sublingual administration of a whole-plant cannabis extract containing THC (range, 30-120 minutes; the mean peak plasma levels were noted 100-130 minutes after administration [higher concentration drops showed a later peak]) (17).

In a study comparing the pharmacokinetics of oral THC with those of THC in a whole-plant cannabis extract (nabiximols), the time to maximal concentration was increased in the latter, although the difference was not statistically significant. Delivery via the oral mucosa resulted in slightly increased bioavailability compared with oral ingestion (18). The bioavailability of THC, in terms of peak plasma level and area under the curve, is increased if the oral mucosal spray is administered during a fed state (19, 20).

In the blood, 90% of THC is distributed to the plasma, and is mainly bound to plasma proteins such as lipoproteins and albumin. Approximately 10% of THC in the blood is distributed in red blood cells. THC rapidly penetrates highly vascularized tissues including the liver, heart, fat, lung, jejunum, kidney, spleen, mammary gland, placenta, adrenal cortex, muscle, thyroid, and pituitary gland. Only approximately 1% of a dose of THC given intravenously is found in the brain at the time when the

psychoactive effects are peaking. Oxidative metabolism of THC yields an active metabolite, 11-hydroxy-delta-9-tetrahydrocannabinol (11-OH-THC). Over time, THC accumulates in less vascularized tissues and finally in body fat, although the exact composition in body fat is not known and may include hydroxyl metabolites and fatty acid conjugates (15).

Cannabidiol: CBD is also highly lipophilic. The absorption and kinetics of CBD from inhaled marijuana smoke have been described as being similar to those of THC, with an average systemic bioavailability of 31% in marijuana smokers (range, 11%-45%) (15). Again, similar to THC, CBD oral bioavailability is poor, (range of 13% to 19%) (12). Peak plasma levels in one study occurred after 1.3 hours (13). Peak plasma levels are similar when CBD is administered as an oral mucosal spray along with THC; however, the time to maximal concentration is longer (18). The bioavailability of CBD, in terms of peak plasma level and area under the curve, is increased if the oral mucosal spray is administered during a fed state (19, 20).

Cannabis for Intractable Pain Management and Interaction with Opioid Medications: Studies of smoked marijuana in patients with pain that was not experimentally induced have concentrated on those with neuropathic pain. In one study, the pain was postsurgical or post-traumatic (21). In other studies, participants had painful human immunodeficiency virus (HIV)-associated sensory neuropathy (22, 23). In all of these studies, smoked marijuana was found to be better than placebo in relieving pain. Another study examined the effects of marijuana that was vaporized (and not smoked) and found that it too was better than placebo for relieving neuropathic pain (patients had central or peripheral neuropathic pain that was resistant to standard treatments) (24). A small study examined the effects of vaporized cannabis on pain in individuals taking extended-release opiates for chronic pain. It found that pain improved with the administration of vaporized cannabis, whereas there was no change in plasma opioid concentrations (25).

Numerous studies (Table 1) (23-31) have explored and reported the effects of smoked marijuana on experimentally induced pain. Smoked marijuana improved pain tolerance in one study (26). In another study, smoked marijuana decreased pain sensitivity and intensity and improved pain tolerance in pain induced by the cold pressor test, in which the participant was instructed to place his or her hand in water at a temperature of 4°C (27). In another study, smoked marijuana had antinociceptive effects based on increased latency of finger withdrawal from radiant heat stimulation compared with placebo (28). In another study, pain was induced by injecting capsaicin intradermally, with medium dose of marijuana reported to decrease pain, whereas a higher dose increased pain (31).

Is there potential for harm with the administration of the constituents of cannabis? Cannabinoids have an extremely favorable drug safety profile (32, 33). Unlike opioid receptors, cannabinoid receptors are not located in brainstem areas controlling respiration, so lethal overdoses due to respiratory suppression do not occur. The administration of cannabinoids to laboratory animals and humans does result in psychoactive effects. In humans, the central nervous system effects are both stimulating and depressing and are divided mainly into four groups: affective (euphoria and easy laughter); sensory (temporal and spatial perception alterations and disorientation); somatic (drowsiness, dizziness, and motor incoordination); and cognitive (confusion, memory lapses and difficulty concentrating) (34).

As cannabinoid receptors are not just located in the CNS but also in tissues throughout the body, additional side effects of note include:

- tachycardia,
- hypotension,

- conjunctival injection,
- bronchodilation,
- muscle relaxation, and
- decreased gastrointestinal motility.

Tolerance to the unwanted side effects of cannabis appears to develop rapidly in laboratory animals and humans. This is reported to occur due to a decrease in the number of total and functionally coupled cannabinoid receptors on the cell surface, with a possible minor contribution from increased cannabinoid biotransformation and excretion with repeated exposure (35).
Although cannabinoids are considered by some to be addictive drugs, their addictive potential is considerably lower than other prescribed agents or substances of abuse. The brain develops tolerance to cannabinoids, with animal research demonstrating a potential for dependence. Dependence is reported to develop in 9% of cannabis users according to the criteria in the DSM-IV (35). The Institute of Medicine report puts this into context, noting that with 46% of the US population ever having used cannabis and 9% becoming dependent, the risk is much lower than that of nicotine, heroin, cocaine, and alcohol, and equivalent to the proportion of those dependent on anxiolytics (34).
Furthermore, we note that a study that investigated the cannabis molecules CBD and THC as allosteric modulators at mu- and delta-opioid receptors (36). The study concluded that CBD was an allosteric modulator of ligand binding to mu- and delta-opioid receptors. Moreover that this pharmacodynamic property was shared by THC. The study reported that all pharmacodynamics effects on the receptors occurred at very high concentrations and could not be expected to contribute to the *in vivo* action of the two molecules / drugs.
Withdrawal symptoms—irritability, insomnia with sleep EEG disturbance, restlessness, hot flashes, and rarely nausea and cramping—have been observed, but are usually mild compared with the withdrawal from opiates or benzodiazepines and usually dissipate after a few days. Unlike other commonly used drugs, cannabinoids are stored in adipose tissue and excreted at a low rate (half-life 1–3 days), so even abrupt cessation of THC intake is not associated with rapid declines in plasma concentration that would precipitate withdrawal symptoms or drug craving (35).
Summary in brief: The controlled medical use of cannabis preparations is currently legal in Austria, Canada, Czech Republic, Finland, Germany, Israel, Italy, the Netherlands, Portugal, and Spain (28). Furthermore, it is estimated that 70% of the US population lives in jurisdictions where they can access medical cannabis. Patients with cancer have a number of symptoms that may be responsive to cannabinoid therapies. As enumerated, these include nausea, vomiting, anorexia, pain, insomnia, anxiety, and depression. Clinicians in current medical practice may have little or no knowledge of the biological actions of endo-cannabinoids and the medicinal qualities of cannabis.

The published literature has often discussed the medicinal use of cannabis, and issues of addiction have dominated with the net effect being that this may have skewed the view of the health consequences of cannabis use by virtue of medical specialty. Practicing oncologists may be more likely to have a much different perception of the risk to benefit ratio of the administration of cannabis compared to other clinicians. There is a suggestion that with an increased and concerted educational effort aimed at healthcare providers, in the coming years medicinal cannabis may become an option for an even larger percentage of patients who may benefit from its use (36).

About the research team and collaboration

The chief investigators comprise a highly–qualified research team with robust collaborative potential. This group is supported and strengthened by research governance from an academic institution and affiliate (industry) that presents a high standard framework of accountability for scientific quality, ethical acceptability, and safety to ensure the delivery of a clinical investigation on the efficacy and safety of a micellized whole cannabis plant derived extract for the management of moderate to severe pain in patients diagnosed with advanced cancer. The research team includes internationally recognised researchers from the fields of epidemiology and clinical trial expertise [PI, AI3, AI5], oncology [PI, AI3, AI5], pain management and clinical trials [AI1, AI2, AI5], pharmacy and clinical pharmacology [AI3] and regulatory aspects of prescribed medicines [AI3, AI4].

The Therapeutic Goods Administration (TGA) and the investigation of cannabis in clinical trials

The proposal provides the HREC with the appropriate scientific and technical expertise relevant to the product that is to be investigated in order to assess safety. As such this determines which scheme the sponsor will utilize to notify the TGA (39). The sponsor will notify the TGA under the Clinical Trial Notification scheme.

Table 1: Effect of smoked or vaporised marijuana on pain (23-31). Adopted from (11).

DESIGN	SUBJECTS	N	HOW ADMINISTERED	CONTROL	TEST	RESULTS
RCT	Both experienced marijuana users and non-users of marijuana	32	Smoke inhaled via smoking device	Placebo	Pain tolerance: pressure algometer (metal rod putting pressure on the thumb)	Increased pain tolerance with marijuana compared with placebo, with a larger effect for experienced users compared with nonexperienced
RCT	Daily marijuana smokers	30	Smoked marijuana and oral THC	Placebo	Cold pressor test	Marijuana and oral THC decreased pain sensitivity, increased pain tolerance, and decreased subjective ratings of pain intensity
RCT	Male regular marijuana users	5	Smoked	Placebo	Finger withdrawal from radiant heat stimulation	Significant dose-dependent antinociception (increased finger withdrawal latency)
RCT	Healthy volunteers	15	Smoked	Placebo	Intradermal capsaicin	No effect with marijuana that was 2% THC by weight, decreased pain with marijuana that was 4% THC by weight, and increased pain with marijuana that was 8% THC by weight
RCT	Adults with posttraumatic or postsurgical neuropathic pain	21	Smoked	Placebo		A single inhalation of 25 mg of 9.4% THC marijuana 3 times daily for 5 d reduced the intensity of pain and improved sleep
RCT/crossover	Adults with HIV-associated distal sensory predominant polyneuropathy refractory to at least 2 previous analgesic classes	28	Smoked	Placebo		Greater pain relief with marijuana than placebo and more subjects had at least 30% pain relief with marijuana compared with placebo (46% vs 18%)
RCT	Adults with painful HIV-associated sensory neuropathy	50	Smoked	Placebo	Brush and von Frey hair stimuli	Marijuana reduced daily and chronic pain more than placebo; also reduced hyperalgesia as measured by brush and von Frey hair stimuli tests
RCT/crossover	Adults with central and peripheral neuropathic pain	39	Vaporized	Placebo		Analgesia with both 3.53% THC-by-weight marijuana and 1.29% THC-by-weight marijuana compared with placebo with no significant difference noted between the doses/concentrations
Single arm	Adults with chronic pain being treated with slow-release opiates	21	Vaporized	None		Pain decreased with no effect on plasma opioid levels

2.2 Research Question

This study will investigate the safety, tolerability and exploratory end-point in a pilot study of a buccally administered THC:CBD extract from *Cannabis sativa* L./Cannabis indica L. as an adjunct to opioid analgesia.

2.3 Rationale for Current Study

Cancer pain is still pervasive and as such can be undertreated in all settings where patients with cancer are managed. This study will assess the safety, tolerability and efficacy of a micellized buccally administered THC:CBD extract in the management of patients with at least moderately severe cancer-related pain despite appropriate pharmacological management.

3 STUDY OBJECTIVES

3.1 Primary Objective

The primary objective is assessment of safety and tolerability by:

- Clinical assessment including: recorded adverse events
- The European Organisation for Research and Treatment of Cancer (EORTC QLQ-C30) Version 3 (see APPENDIX 1, pages 41 - 44)
- Laboratory assessments including pharmacokinetic data

3.2 Secondary Objectives

The secondary objective is to assess Analgesic efficacy as assessed by:

- Mean pain Numerical Rating Scale (NRS) scores (Please see APPENDIX 2 and 3, pages 43 and 44) from the participant diary symptom scores and investigator-assessed pain control scores.
- Rescue analgesia (opioid analgesic) doses

4. STUDY DESIGN

4.1 Type of Study

A study investigating the safety, tolerability and exploratory end-point pilot study of a buccal administered cannabis extract (THC:CBD) as an adjunct to opioid analgesics for the management of moderate to severe pain in patients diagnosed with advanced cancer.

4.2 Study Design

The study has 2 stages:

1. Stage 1: Pharmacokinetic arm, please see Table 2 on page 30.
2. Stage 2: Dose-Escalation-Treatment Phases, please see Figure 1 and Table 3 pages 32-33.

The study will commence following NSLHD HREC submission approval and CTN approval from the

TGA.

STAGE 1 (n= 5 participants with advanced cancer) Pharmacokinetic Phase of the study:

Day -2:

- Completion of signed informed consent
- Potential participants to attend RNSH for physical examinations, blood collection and on satisfying inclusion and exclusion criteria
- Participants will be informed that they will be hospitalised for 48 hours [2 nights].

Day 1:

- Open label single treatment group pharmacokinetic arm of the study administering 2 actuations of the pump (delivering 2.6 mg THC+2.4 mg CBD in 0.3 mL = one standard dose)
- Participants admitted into RNSH
- Dose administration approximately 9:30AM
- Blood collections at 0, 30, 60, 90, 120, 150, 180, 240, 360 minutes and at 12 and 24 hours

Day 2 (consecutive):

- Administering 6 actuations of the pump (delivering 7.8 mg THC+7.2 mg CBD in 0.9 mL = 3 standard doses)
- Dose administration approximately 9:30AM
- Blood collections at 0, 30, 60, 90, 120, 150, 180, 240, 360 minutes and at 12 and 24 hours
- Note: that the PI will oversee the patient admitted for two nights (in stage 1) and may keep the patient in hospital if deemed relevant beyond the mandatory admission of 2 nights based on any adverse changes from the investigational drug.

DSMB to review data before continuation to the next stage.

STAGE 2 (n=25 participants with advanced cancer and intractable pain not responsive to opioid medications): Dose Escalation- Treatment Phases of the study.

Consists of (i) dose escalation phase; (ii) dose treatment phase; (iii) follow-up phase.

Day -2 RNSH:

- Completion of signed informed consent.
- Potential participants to attend RNSH for physical examinations, blood collection and on satisfying inclusion and exclusion criteria.
- Complete of questionnaires (BPI-SF, EORTC QLQ-C30) and NRS Score
- Participants will be informed that they will be admitted to the hospital for one night initially at each of the dose escalation phases of stage 2 namely on days 1, 4 and 7. See Table 3 on page 32.
- Note: that the PI will oversee the patient admitted to RNSH on Days 1, 4 and 7 for one night at each of the dose escalation phases of stage 2 and may keep the patient in hospital for a longer period of time if deemed relevant beyond the mandatory admission (1 night) based on any adverse changes from the investigational drug. Please see Table 3 on page 32.

Day 1 (Start Dose-Escalation Phase) RNSH:

- Participants attend and admitted to RNSH for 1 night.
- The Research Nurse will provide a) advice on dosing b) instruct the patient of the dose that should be administered and c) the treatment clinician will be advised and be part of this communication process.
- Dispense new cannabis vial for 3 days of dosing only (limited supply).
- Participants demonstrated on how to administer cannabis by the Research Nurse.
- Participants (under supervision) administers first standard dose (2 actuations of the pump delivering 2.6 mg THC+ 2.4 mg CBD in 0.3 mL) and then continues to self-administer at RNSH and at home every 4 hours unless asleep and repeat for the next 2 days.
- Participants collect and instructed how to complete case report form (CRF)

Day 2 Home:

- The Research Nurse will provide a) advice on dosing b) telephone daily to remind the patient of the dose that should be administered and c) the treatment clinician will be advised and be part of this communication process.
- Participants administer at home a standard dose of 2 actuations of the pump (delivering 2.6 mg THC+2.4 mg CBD in 0.3 mL) every 4 hours unless asleep.
- Participants to complete questionnaires (BPI-SF, EORTC QLQ-C30), CRF and NRS Score at the end of the day

Day 3 Home:

- The Research Nurse will provide a) advice on dosing b) telephone daily to remind the patient of the dose that should be administered and c) the treatment clinician will be advised and be part of this communication process.
- Participants administer at home a standard dose of 2 actuations of the pump (delivering 2.6 mg THC+2.4 mg CBD in 0.3 mL) every 4 hours unless asleep.
- Participants to complete questionnaires (BPI-SF, EORTC QLQ-C30), CRF and NRS Score at the end of the day.

Day 4 RNSH:

- Participants return to RNSH and admitted for 1 night.
- Physical examinations and blood collection (at approx. 9 am).
- Participants to present completed CRF to Research Nurse.
- Return used cannabis vial and dispense new vial for 3 days of dosing (limited supply).
- The Research Nurse and PI will provide a) advice on dosing b) instruct the patient of the dose that should be administered and c) the treatment clinician will be advised and be part of this communication process.
- PI to approve an increased dose to two standard doses (4 actuations of the pump delivering 5.2 mg THC + 4.8 mg CBD in 0.6 mL) every 4 hours unless asleep.
- Participants to complete questionnaires (BPI-SF, EORTC QLQ-C30), CRF and NRS Score at the end of the day

Days 5 Home:

- The Research Nurse will provide a) advice on dosing b) telephone daily to remind the patient of the dose that should be administered and c) the treatment clinician will be advised and be part of this communication process.
- Participants administer at home two standard doses (4 actuations of the pump delivering 5.2 mg THC + 4.8 mg CBD in 0.6 mL) every 4 hours unless asleep.

- Participants to complete questionnaires (BPI-SF, EORTC QLQ-C30), CRF and NRS Score at the end of the day,

Day 6 Home:

- The Research Nurse will provide a) advice on dosing b) telephone daily to remind the patient of the dose that should be administered and c) the treatment clinician will be advised and be part of this communication process.
- Participants administer at home two standard doses (4 actuations of the pump delivering 5.2 mg THC + 4.8 mg CBD in 0.6 mL) every 4 hours unless asleep.
- Participants to complete questionnaires (BPI-SF, EORTC QLQ-C30), CRF and NRS Score at the end of the day

Day 7 RNSH:

- Participants return to RNSH and admitted for 1 night.
- Patients undergo physical examinations and blood collection (at approx. 9am).
- Participants to present completed CRF to Research Nurse
- Return used cannabis vial and dispense new vial for second phase of dose escalation phase of stage 2 (limited supply).
- The Research Nurse will provide a) advice on dosing b) instruct the patient of the dose that should be administered and c) the treatment clinician will be advised and be part of this communication process.
- Research Nurse and PI to approve increased to three standard doses (6 actuations of the pump delivering 7.8 mg THC + 7.2 mg CBD in 0.9 mL) every 4 hours unless asleep repeat during 1 day while in hospital and for 2 days at home.
- Participants to complete questionnaires (BPI-SF, EORTC QLQ-C30), CRF and NRS Score at the end of the day.

Day 8 Home:

- The Research Nurse will provide a) advice on dosing b) telephone daily to remind the patient of the dose that should be administered and c) the treatment clinician will be advised and be part of this communication process.
- Participants to administer at home three standard doses (6 actuations of the pump delivering 7.8 mg THC + 7.2 mg CBD in 0.9 mL) every 4 hours unless asleep.
- Participants to complete questionnaires (BPI-SF, EORTC QLQ-C30), CRF and NRS Score at the end of the day.

Day 9 Home:

- The Research Nurse will provide a) advice on dosing b) telephone daily to remind the patient of the dose that should be administered and c) the treatment clinician will be advised and be part of this communication process.
- Participants to administer at home three standard doses (6 actuations of the pump delivering 7.8 mg THC + 7.2 mg CBD in 0.9 mL) every 4 hours unless asleep.
- Participants to complete questionnaires (BPI-SF, EORTC QLQ-C30), CRF and NRS Score at the end of the day.

Day 10 (start Treatment Phase) RNSH/Home:

- Participants attend RNSH for physical examinations and blood collection (at approx. 9am).
- Return used cannabis vial and dispense new cannabis vial for the ongoing treatment phase for 3 days of dosing (limited supply).

- Self-administer at home the prescribed dose* once every 4 hours unless asleep. **[the prescribed dose of the cannabis is that dose defined by safe and tolerable administration of the cannabis medicine that improves the management of pain, as defined and directed by the PI*]
- Participants to present completed CRF to Research Nurse.
- Participants to complete questionnaires (BPI-SF, EORTC QLQ-C30), CRF and NRS Score at the end of the day.
- The Research Nurse will provide a) advice on dosing b) telephone daily to remind the patient of the dose that should be administered and c) the treatment clinician will be advised and be part of this communication process.

Day 11 Home:

- The Research Nurse will provide a) advice on dosing b) telephone daily to remind the patient of the dose that should be administered and c) the treatment clinician will be advised and be part of this communication process.
- Participants administer at home the prescribed dose once every 4 hours unless asleep.
- Participants to complete questionnaires (BPI-SF, EORTC QLQ-C30), CRF and NRS Score at the end of the day.

Day 12 Home:

- The Research Nurse will provide a) advice on dosing b) telephone daily to remind the patient of the dose that should be administered and c) the treatment clinician will be advised and be part of this communication process.
- Participants administer at home the prescribed dose once every 4 hours
- Participants to complete questionnaires (BPI-SF, EORTC QLQ-C30), CRF and NRS Score at the end of the day

Day 13 RNSH/Home:

- Participants attend RNSH for physical examinations and blood collection (at approx. 9am).
- Participants to present completed CRF to Research Nurse.
- Return used cannabis medicine vial.
- Dispense new cannabis vial for 3 days of dosing (limited supply).
- The Research Nurse will provide a) advice on dosing b) telephone daily to remind the patient of the dose that should be administered and c) the treatment clinician will be advised and be part of this communication process.
- Participant administer at home prescribed dose once every 4 hours unless asleep.
- Participants to complete questionnaires (BPI-SF, EORTC QLQ-C30), CRF and NRS Score.

Day 14 Home:

- The Research Nurse will provide a) advice on dosing b) telephone daily to remind the patient of the dose that should be administered and c) the treatment clinician will be advised and be part of this communication process.
- Participants administer at home the prescribed dose once every 4 hours unless asleep.
- Participants to complete questionnaires (BPI-SF, EORTC QLQ-C30), CRF and NRS Score at the end of the day.

Day 15 Home:

- The Research Nurse will provide a) advice on dosing b) telephone daily to remind the patient of the dose that should be administered and c) the treatment clinician will be advised and be part of this communication process.

- Participants administer at home the prescribed dose once every 4 hours unless asleep. This is the last day of dose administration that the participant takes for this study.
- Participants to complete questionnaires (BPI-SF, EORTC QLQ-C30), CRF and NRS Score at the end of the day.

Day 16 (start Follow-up Phase) RNSH:

- Participants attend RNSH for physical examinations and blood collection (at approx. 9am).
- Return used cannabis medicine vial at RNSH with Research Nurse.
- Return completed CRF at RNSH with Research Nurse.

Day 30 (Final Day) RNSH:

- Participants attend RNSH for physical examinations and blood collection (at approx. 9am).
- Participants to complete questionnaires (BPI-SF, EORTC QLQ-C30) and NRS Score.

<u>DSMB second meeting to review all data before completing report.</u>

Please note that:

1 Participants will be instructed by a Research Nurse on how to administer the buccal mucosa THC:CBD cannabis medicine for this open label single treatment group allocation study.

2 Participants will be hospitalized on days 1, 4, 7, and reviewed on days 10, 13, 16 and 30 of stage 2.

3 Participants will complete a study diary (found in individual participants CRF) and record pain scores three times per day and background medication and all additional breakthrough analgesia on each day of participation in Stage 2. During the visits days at RNSH a delegate of a pain specialist as required may also assess the level of pain experienced by the participant.

4 Also blood pressure, heart rate, respiration, and temperature will be recorded at RNHS visits.

5 Tolerability to the cannabis medicine (e.g., any adverse psychosis events that may eventuate) will also be recorded at the RNSH visits.

6 Medications (including all analgesics and analgesic adjuvants) administered will be recorded on all days of the Stage 2 participation.

7 During the dose escalation-treatment phases of Stage 2 of the study participants will be asked to complete a study diary (CRF) daily until the end of the dose escalation-treatment phases of the study.

8 During Stage 2 eligible participants will measure daily their quality of life by filling in the European Organization for Research and Treatment of Cancer (EORTC QLQ-C30) Version 3 questionnaire (see appendices this document and CRFs).

9 The study medication that will be delivered will use a pump action buccal fine mist spray.

10 If unwanted effects develop on a new number of actuations (sprays), the participant will not take any further actuations (sprays)

    a. As patients with advanced cancer are tracked, it is expected their routine medications will be adjusted, such as, the administration of palliative radiotherapy, chemotherapy and in some cases nerve blocks.

<u>DSMB second meeting to review all data before completing report.</u>

4.3 Number of Participants

30

4.4 Study sites

Royal North Shore Hospital

4.5 Expected Duration of Study

9 months

4.6 Primary and Secondary Outcome Measures

The primary objective is assessment of safety and tolerability by:

- Clinical assessment including: recorded adverse events
- The European Organisation for Research and Treatment of Cancer (EORTC QLQ-C30) Version 3 (see APPENDIX 1, page 38 - 39)
- Laboratory assessments including pharmacokinetic data

The secondary objective is to assess Analgesic efficacy as assessed by:

- Mean pain Numerical Rating Scale (NRS) scores (SEE APPENDIX 2 and 3, pages 41 and 42) from the participant diary symptom scores and investigator-assessed pain control scores. Rescue analgesia (opioid) doses

5. STUDY TREATMENTS

5.1 Treatment Arms

5.1.1 Description

The study medication that will be delivered will use a pump action of a fine mist spray of a micellized THC:CBD extract (Refer to *Investigator Brochure* for details).

5.1.2 Dosage and Route of Administration

STAGE 1:
DAY 1: Administer to the buccal mucosa site one standard dose (2 actuations of the pump delivering 2.6 mg THC+2.4 mg CBD in 0.3 mL = one dose) on study day 1.
DAY 2 consecutive: Administer to the buccal site three standard doses (6 actuations of the pump delivering 7.8 mg THC+7.2 mg CBD in 0.9 mL = 3 doses) at

STAGE 2:
Dose escalation phase of the study:

- Administer to the buccal mucosa site one standard dose (2 actuations of the pump delivering 2.6 mg THC+2.4 mg CBD in 0.3 mL = one dose) on days 1-3, every 4 hours unless asleep.

- Administer to the buccal mucosa site two standard doses (4 actuations of the pump delivering 5.2 mg THC+4.8 mg CBD in 0.6 mL) on days 4-6, every 4 hours unless asleep.
- Administer to the buccal mucosa site three standard doses (6 actuations of the pump delivering 7.8 mg THC+7.2 mg CBD in 0.9 mL) on days 7-9, every 4 hours unless asleep.

Treatment phase of the study: patients will administer to the buccal mucosa site either one, two or three doses every 4 hours (unless asleep) for 7 days. The dose to be administered will be dependent on the safe and tolerable dose administered in the previous dose escalation phase of the study, as directed by the PI.

5.1.3 Dose modification

N/A

5.2 Preparation and administration of study drug

5.2.1 Preparation

Pharmaceutical Packaging Professionals (PPP) [TGA licensed (ID: 50963 / License number: MI-2013-LI-07674-1) manufacturer] will:

- Be responsible for finished product manufacturing, primary packaging and labelling, release of product for clinical trial and warehousing and distribution.
- Hold a current Commonwealth import and export licence and the Victorian Drugs Poisons and Controlled Substances Licence for manufacture, warehousing and distribution of S8 products.
- Notify individual state DPCS departments of the arrangement for distribution of study drug.
- Handle and transport S8 drugs to sites using World Couriers. The same arrangement is in place for ongoing CINV studies at RNSH.
- Obtain from Aptar the international leading manufacturer of spray pumps for the pharmaceutical industry the pumps for the study. Spray pumps are not TGA approved items (container and packaging materials). The pumps will be tested on receipt against the manufacturers specification to confirm uniformity of shot volume (based on weight) and reproducibility of shot volume.
- Utilise World Couriers (transport services), to operate secure tamper evident boxes with temperature loggers. All shipments will be sent with packaging slips. No external indicator of contents will be included on the containers.

5.2.2 Administration

- A Research Nurse will first show patients how to administer the drug with the pump spray.

- In Stage 1 participants are under 48 hours of observation.
- In Stage 2 participants admitted to overnight stay to RNSH on mandatory days 1, 4 and 7 and visit RNSH on days 10, 13, 16 and 30.
- PI is responsible for ALL dose escalations.

Dispensing and Product Accountability

The medicine will be accounted for by the Royal North Shore Hospital Pharmacy. (Note: RNSH pharmacy will stock management, dispensing and control of all of the investigational drug vials as well as ensure drug storage and environmental monitoring under drug S8 conditions). The cannabis product will not require refrigeration.

5.3 Measurement of participant compliance

- Participant compliance will be measured from participant diaries and exploring the accurate reporting, interpretation verification and validity of the recorded data and that of missing data.
- Maintain and review
  - Trial Master Files
  - Investigator Site Files
  - CRFs and Source diary documents

5.4 Excluded medications and treatments

Levodopa, Sildenafil (or any other PDE5s are excluded on safety grounds) and any hypersensitivity to cannabinoids.

*The administration of medications that may affect psychoactive states are not excluded as the use of sedation for anxiety/depression is dependent on how the patients are tracking.

6. PARTICIPANT ENROLMENT

6.1 Recruitment

A research study coordinator will review files of inpatients and suitable outpatients and identify those patients that have been diagnosed with advanced cancers with intractable pain and as such could potentially be inclined to participate in the pharmacokinetic (Stage 1) or dose escalation–treatment-follow-up phases (Stage 2) of the study. Participants who are inclined to participate in the pharmacokinetic study will be also eligible to participate in the dose escalation–treatment-follow-up stage of the study if they so wish if they become opioid non-responsive.
The following initial criteria will govern whether a potential participant could be followed-up for screening:

- Adult male or female participants who have been using strong opioids for at least one week to relieve pain associated with incurable malignancy and who have given written

informed consent will be screened for study entry.

6.2 Eligibility Criteria

6.2.1 Inclusion Criteria Stage 1

1. Patient aged greater than 18 years.
2. Patient able to give informed consent and comply with study procedures.
3. Patient that has been diagnosed with an incurable malignancy.
4. Patient willing and able to receive an oro-buccal mucosa delivered medicine.
5. Patient willing to take a medication, which may exhibit psychoactive effects.
6. Patient agrees to abstain from using cannabis medicines other than the experimental drug for the duration of the clinical trial.
7. Patient agrees to not partake in any other interventional clinical treatments other than the ones that the treating clinical team are already aware of for the duration of the study.

8. Patient consents to provide blood samples for analysis throughout the duration of the clinical trial, whether participation is for Stage 1 only and or Stage 2.
9. Patient agrees to disclose that they are receiving an experimental medication to treating medical professionals who might be unaware of this fact.
10. Female of childbearing potential, agrees to use an effective form of birth control during the study participation.
11. Patient consents to having a baseline test for pregnancy if applicable.
12. Patient consents to having baseline tests for recent illicit substance use.
13. Patient agrees to still take regular doses of an opioid analgesic as prescribed. Patient should not cease opioid medications.

6.2.2 Inclusion Criteria Stage 2

1. Patient aged greater than 18 years.
2. Patient able to give informed consent and comply with study procedures.
3. Patient that has been diagnosed with an incurable malignancy.
4. Patient reported experiencing moderate to severe pain.
5. Patient has been using strong opioid analgesics for at least one week to relieve pain associated with incurable malignancy. One week prior use of opiate treatment is sufficient duration because it would represent established opioid treatment and most patients would have developed tolerance after one week, especially if it is given around the clock at a total daily dose of at least 60 mg of oral morphine or equivalent.
6. Patient report pain severity that is scored to be greater than a rating of 4 within a 0-10 Numerical Rating Scale (NRS) assessment tool.
7. Patient willing and able to receive an oro-buccal mucosa delivered medicine.
8. Patient willing to take a medication, which may exhibit psychoactive effects.
9. Patient agrees to abstain from using cannabis medicines other than the experimental drug for the duration of the clinical trial.
10. Patient agrees to not partake in any other interventional clinical treatments other than the ones that the treating clinical team are already aware of for the duration of the study.

11. Patient consents to provide blood samples for analysis throughout the duration of the clinical trial, whether participation is for Stage 1 only and or Stage 2.
12. Patient agrees to disclose that they are receiving an experimental medication to treating medical professionals who might be unaware of this fact.
13. Female of childbearing potential, agrees to use an effective form of birth control during the study participation.
14. Patient consents to having a baseline test for pregnancy if applicable.
15. Patient consents to having baseline tests for recent illicit substance use.
16. Patient agrees to still take regular doses of an opioid analgesic as prescribed. Patient should not cease opioid medications.

6.2.3 Exclusion Criteria

1. Patient engaged in medicinal or recreational use of any cannabinoid containing substance, in any form within the past 30 days.
2. Patient has a cognitive impairment or intellectual disability.
3. Patient has a history of primary psychotic disorder, bipolar affective disorder, bipolar disorder with psychotic features, depressive disorder with psychotic features, borderline personality disorder, antisocial personality disorder, or a positive family history (first degree relative) of psychotic disorder or bipolar affective disorder.
4. Patient has any history of allergic or hypersensitivity reaction to any herbal product, including cannabinoids.
5. Patient reports a prior sensitivity reaction to an oral-mucosal administered medicine or supplement (e.g., Liposomes).
6. Patient has been diagnosed with a head, neck, or oral-pharyngeal cancer.
7. Patient has undergone any radiotherapy to the mouth or oral cavity.
8. Patient presents with any cardiovascular disorders (Coronary artery disease; heart attack; abnormal heart rhythms or arrhythmia; heart failure; heart valve disease; congenital heart disease; heart muscle disease; pericardial disease; aorta disease; vascular disease), epilepsy (or a previous history of seizures), psychoactive disorders or any clinically significant hepatic or renal impairment.
9. Patient has been diagnosed with brain metastasis.
10. Patient is pregnant, lactating or partaking in sexual contact, which may result in pregnancy without adequate contraception.
11. Patient has received epidural analgesia within 48 hours of the baseline assessment
12. Patient has received any radiotherapy within two weeks of the initial baseline assessment.
13. Patient is currently not taking Levodopa, Sildenafil (or any other PDE5s), anticonvulsants and/or Cannabinoids
14. Patient is currently receiving ketamine.

6.3 Informed Consent Process

There are 2 stages to this investigational study. Stage 1: A potential participant will be identified from the RNSH records of patients undergoing treatment for a cancer diagnosis. Stage 2: A

potential participant will be identified from the RNSH records of patients undergoing treatment for a cancer diagnosis and with intractable pain. The PK investigation (Stage 1) will be conducted first and completed prior to commencing the dose escalation–interventional phase (Stage 2). Given that the participants for both stages are the same type of patients namely diagnosed with advanced cancer and with intractable pain, and potentially be a participant in both stages of the investigation, the informed consent process will follow the same procedures.

The entire informed consent process will involve giving a prospective participant:

- Clear and adequate information concerning the phase of the study that is about to begin, namely the PK or the dose escalation–interventional stage,
- Providing adequate opportunity for the participant to consider all options,
- Responding to the participant's questions,
- Ensuring that the participant has understood all information,
- Obtaining the participant's voluntary agreement to participate and,
- Continuing to provide information as the subject or situation requires.
- Documentation that clearly states they are participating in a clinical trial and this will be clearly outlined in the appropriate and relevant PISCF for the PK stage 1-study or the PISCF for the Stage 2 dose escalation–interventional study.

A research Co-ordinator / Nurse will be responsible for administering participant consent forms and PISCF.

The following line will be adopted for an effective discourse between participant and investigator:

1. The consent document will be used as a guide to explain the study to a prospective participant.
2. The consent document will constitute the basis for a meaningful verbal exchange between the prospective participant and the research investigator and this document will NOT serve as a substitute for discussion.
3. The process will provide ample opportunity for the Investigator and the subject to exchange information and ask questions.
4. Any questions relevant to the study and its procedures arising from the exchange will be clearly detailed to the participant prior to seeking consent.
5. Once all queries have been answered and study procedures understood by the participant, documentation will be provided to the participant and a written consent form containing all the information to be disclosed and signed by the participant will be obtained and a copy provided to the participant.

6.4 Enrolment and Randomisation Procedures

Participants identified as eligible by meeting all inclusion and exclusion criteria to participate and who have subsequently completed and signed an informed consent will be enrolled into the single arm study. The participant will receive a study enrolment number and this will be documented in the participant’s medical record and on all study documents.

6.5 Blinding Arrangements

Open label (not blinded)

6.6 Breaking of the Study Blind

6.6.1 On Study

Not applicable

6.6.2 Following Completion of the Study

Not applicable

6.7 Participant Withdrawal

6.7.1 Reasons for withdrawal

Taking a harm minimization/best interest approach, removing a patient from the clinical study will be at the clinical discretion of PI Clarke in consultation with other members of the research team and the treating clinician and if agreed upon PI Clarke may remove any participant from the clinical study at any time if:

1. In the event a participant's safety may be compromised (e.g., serious adverse event(s) or unanticipated health problems;
2. The study is terminated by the investigator or sponsor related to increased risk to participants;
3. The participant is non-compliant (e.g., patient health deteriorates; patient fails to administer the drug; patient does not comply with dose administration) with the study protocol / procedures;
4. The PI determines that it is in the best interest of the participant to be removed from the study.

6.7.2 Handling of withdrawals and losses to follow-up

The PI Clarke may remove a research participant from the study at any time in the event the participant's safety may be compromised such as the following:

- any unanticipated health problems;
- the study is terminated by the investigator or sponsor related to increased risk to participants;
- the participant is non-compliant with the study protocol / procedures;
- PI determines that it is in the best interest of the participant to be removed from the study.

Note: Criteria for participant removal by the PI Clarke will be outlined in the informed consent.

6.7.3 Replacements

Participant replacement due to withdrawal will be assessed on an individual basis and reason for withdrawal.

6.8 Trial Closure

Relevant to investigator(s):

1. Provide a summary report of the trial's outcome to the ethics committee as required.
2. Keep documentation and correspondence in the trial master file in accordance with GCP.
3. Inform the sponsor of the completion of the study.
4. Ensure arrangements for archiving of trial documents are clarified.
5. Ensure appropriate final disposition of any investigational product, and this may include return to the sponsor or destruction of remaining materials.

Relevant to Participants:

Participants will be followed up for the development of adverse events for one month following completion of the clinical study.

6.9 Continuation of therapy

In the event that the medication is effective over the two-week period, Medlab will make available the medication to all trial participants free of charge in consultation with their treating physician, after the completion of the study. The patient's clinician will be informed of participation (dosage and response / tolerance and of any AEs) and that on completion of the study if the patient wishes to continue usage of the cannabis medicine.

It will also be emphasised in the participant information sheet that there will be no legal issue in obtaining the product from Medlab. The product that is provided by Medlab, tentatively labelled as NanaBis™, is a cannabis pharmaceutical product and should not be considered as medicinal marijuana.

7. <u>STUDY VISITS AND PROCEDURES SCHEDULE</u>

Table 2: Stage 1 Pharmacokinetic studies procedures.

List Interventions	Pre Enrolment (Visit Day -2)	Start Stage 1 PK study Day 1*	Start Stage 1 PK study Day 2*
Inclusion / Exclusion criteria	✓		
Informed Consent	✓		
Physical examination	✓		
NRS score<br>BPI-SF	✓<br>✓	✓<br>✓	✓<br>✓
EORTC<br>QLQ-C30	✓<br>✓	✓<br>✓	✓<br>✓
Administer cannabis medicine		Clinical Trial coordinator will instruct dose administration and correct use of the cannabis product.	Clinical Trial coordinator will instruct dose administration and correct use of the cannabis product.

Collect Blood samples (5mL) for cannabinoid PK analysis at time points as indicated<br><br>Blood sample (5mL) collected for safety assessment RNSH lab.	✓<br><br>✓	1 dose (2 actuations) will be administered at 0 minutes.<br><br>Baseline collections at 0, 30, 60, 90, 120, 150, 180, 240, 360 minutes and at 12 and 24 hours.<br><br>At 24 hours	3 doses (6 actuations 2 minutes apart) starting at 0 minutes.<br><br>Blood collections at 0, 30, 60, 90, 120, 150, 180, 240, 360 minutes, and at 12 and 24 hours.<br><br>At 24 hours.
Adverse Events and Serious Adverse Events		✓	✓

* Participants will be admitted to hospital for two nights initially in this stage 1 of the study.

Note: that the PI will oversee the patient admitted to RNSH on both days and may keep the patient in hospital for a longer period of time if deemed relevant beyond the mandatory admission (2 nights) based on adverse changes from the investigational drug.

Figure 1: Dose escalation Stage 2 processes

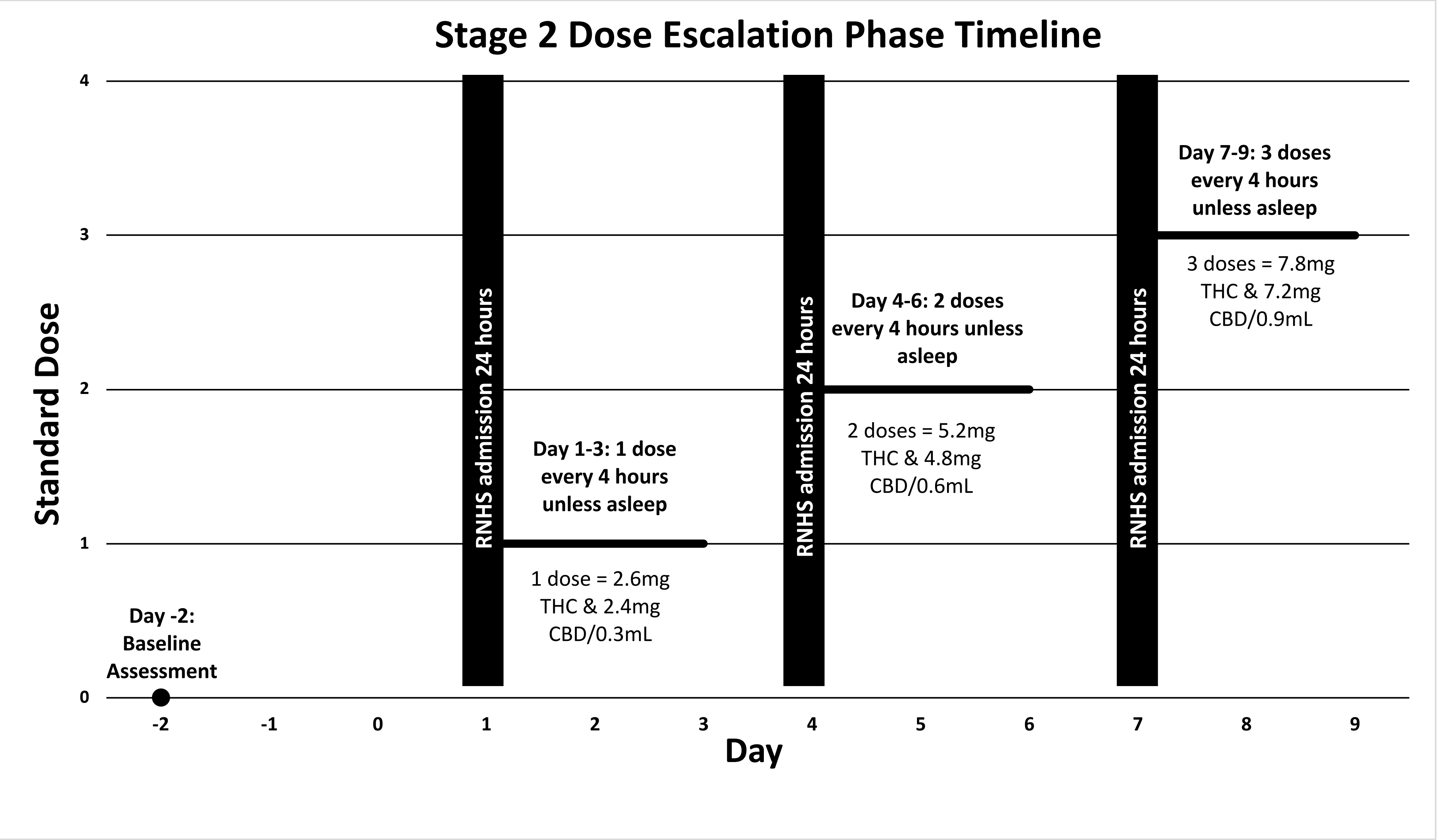

Table 3: Stage 2 dose escalation-treatment-follow-up procedures

		Pre Enrolment (Visit Day)	Stage 2 Dose Escalation Phases (Period)									Stage 2 Treatment Phase (Period)						Follow Up Post Treatment Phase / Treatment Cessation		
			Phase-1 1 Dose			Phase-2 2 Doses			Phase-3 3 Doses			Established Dose								
Site	Task \ Day	-2	1 *	2	3	4 *	5	6	7 *	8	9	10	11	12	13	14	15	16		30
RNSH	Informed Consent	✓																		
RNSH	Inclusion / Exclusion Criteria	✓																		
Home /RNSH	NRS Score	✓		✓	✓	✓	✓	✓	✓	✓	✓	✓	✓	✓	✓	✓	✓			✓
Home /RNSH	BPI-SF	✓		✓	✓	✓	✓	✓	✓	✓	✓	✓	✓	✓	✓	✓	✓			✓
Home /RNSH	EORTC QLQ-C30	✓		✓	✓	✓	✓	✓	✓	✓	✓	✓	✓	✓	✓	✓	✓			✓
RNSH	Drug Dispensing		✓			✓			✓			✓			✓					
RNSH	Physical Examination	✓				✓			✓			✓			✓			✓		✓
RNSH	Blood Sample (15mL) Collection	✓				✓			✓			✓			✓			✓		✓
RNSH /Home	Adverse Events	✓				✓			✓			✓			✓			✓		✓
RNSH/ Home	Serious Adverse Events					✓			✓			✓			✓			✓		✓

* Participants will be admitted to hospital for one night initially at each phase of stage 2 dose escalation namely on days 1, 4 and 7.

<u>Please note:</u> that the PI will oversee the patient admitted to RNSH on Days 1, 4 and 7 for one night of the dose escalation phase of stage 2 and may keep the patient in hospital for a longer period of time if deemed relevant beyond the mandatory admission (1 night) based on adverse changes from the investigational drug.

8. CLINICAL AND LABORATORY ASSESSMENTS

Eligible participants will provide blood samples at different time points as outlined in section 7 Tables 2 and 3 for the pharmacokinetic and dose escalation–treatment-follow-up phases of the study Stages 1 and Stage 2 respectively. Blood samples will be collected as described in Tables 1 and 2 on the designated days for the PK (Stage 1) and dose escalation–treatment-follow-up phases (Stage 2) of the study. Note that blood samples collected in Stage 2 part of the study are research specific to enable the further detection of THC and CBD in blood that allows researchers to inform on the exploratory end-point part of the pilot study.

9. ADVERSE EVENT REPORTING

Adverse event reporting for clinical trials involving therapeutic products, must meet the requirements of the National Health and Medical Research Council, Australian Health Ethics Committee (AHEC) Position Statement "*Monitoring and reporting of safety for clinical trials involving therapeutic products*" (May 2009), which can be found at:

https://www.nhmrc.gov.au/guidelines-publications/e112

9.1 Definitions

An adverse event for medicines is also referred to as an adverse experience, any untoward medical occurrence in a patient or clinical investigation participant administered a pharmaceutical product and which does not necessarily have a causal relationship with this treatment.

An adverse event can therefore be any unfavourable or unintended sign, symptom or disease temporarily associated with the use of a medicinal (investigational) product, whether or not related to the medicinal (investigational) product.

Clinically significant liver dysfunction will be defined by 2.5 times higher abnormal liver function tests (in the absence of hepatic metastasis); and clinically significant kidney dysfunction will be defined by 1.5 times decrease in Glomerular Filtration Rate (GFR of <50); and relevant to the full blood count a white blood cell count > 1,500 cells / µL and platelets >100,000 cells / µL.

9.2 <u>Assessment and Documentation of Adverse Events</u>

For this clinical study, an adverse event (AE) is defined as any unfavourable and unintended change in the:

- signs, symptoms or chemistry (abnormal lab result) that the participant experiences temporarily associated with the use of the sponsor's product, whether or not considered related to the use of the product;
- worsening of a pre-existing condition or symptom.

The assessment will be undertaken by a physician investigator with Research Co-ordinator / Nurse assistant to the PI will undertake to record the information experienced by the participant including:

- all laboratory data / results (e.g. raised Gama GT, hypocalcaemia)
- all adverse experiences such as reported from common cannabis treatment related adverse events, including most mild to moderate severity AEs, psychosis, somnolence, dizziness, confusion, vomiting, hypotension, blurred vision, drowsiness, dry eyes, visual hallucinations, relaxation, coordination disturbance, euphoria, headache, and nausea

9.3 Eliciting Adverse Event Information

Eliciting adverse event information of drug harm and tolerability rely, in part, on clinical trial participant reports of adverse events (AEs), medical histories and concomitant medications.

Recognize:

- the difference between a non-serious adverse event and a serious adverse event
- a suspected adverse reaction
- unexpected or unanticipated adverse event

Differentiate:

- between severity and serious adverse event

9.4 Serious Adverse Event Reporting

9.4.1 SAEs

Serious adverse event (SAE):

An unforeseen medical event that occurs in the course of clinical research that:

- results in participant death.
- is life-threatening to the participant.
- requires the inpatient hospitalisation or prolongation of existing hospitalisation for the participant leads to the participant having a persistent or significant disability/incapacity.

For medicines, also referred to as serious adverse drug reaction, any untoward medical occurrence that at any dose:

- results in death;
- is life-threatening;
- requires in-patient hospitalisation or prolongation of existing hospitalisation;
- results in persistent or significant disability/incapacity;
- is a congenital anomaly/birth defect; or
- is a medically important event or reaction.

NOTE: The term 'life-threatening' in the definition of 'serious', refers to an event in which the patient was at risk of death at the time of the event; it does not refer to an event/reaction which hypothetically might have caused death if it were more severe.

9.4.2 SUSARs

Suspected Unexpected Serious Adverse Reaction (SUSAR)

All adverse events that are suspected to be related to an investigational medicinal product and that are both unexpected and serious are considered to be SUSARs.

A serious adverse event for which there is some degree of probability that the event is an adverse reaction to the administered drug and the adverse reaction is unexpected.

Serious event NOT outlined in the study protocol or information sheet.

9.5 Specific Safety Considerations (e.g., Radiation, Toxicity)

Eligible participants will be monitored for AEs associated with increased risk of developing psychosis due to the administration of the cannabis medicine.

10 STATISTICAL METHODS

10.1 Sample Size Estimation

This is a safety, tolerability and exploratory end-point pilot study that has 2 Stages.
Stage 1: PK n=5 with patients diagnosed with advanced cancers.
Stage 2: Dose escalation–treatment phases n=25 with patients diagnosed with advanced cancers and intractable pain (i.e., unresponsive to opioid medications).
Sample size calculation for the dose escalation–treatment phase of the study was assigned a power of 80% for a moderate effect size of 0.5 and a significance of 0.05 (one tail) and this gave a sample size of n=25.
The total sample size for this study will be n=30

10.2 Population to be analysed

All eligible participants that have participated for at least 1 dose will be included in an intention to treat analysis.

10.3 Statistical Analysis Plan

For the primary outcome of safety and tolerability the results will be descriptive and quantitative. For the two secondary efficacy variables (i.e., NRS pain score and use of breakthrough medication), the Hochberg (3) method will be used to test the global hypothesis for a treatment effect on pain. The daily pain NRS score will be the mean of all the daily assessments. The change in mean NRS pain scores from baseline (all days in run-in period) to the end of treatment will be analyzed using analysis of covariance (ANCOVA), with baseline pain as a covariate and grouped study and treatment as factors (4,5). The proportions of responders (patients with >= 30% improvement from baseline to end of study NRS and BPI-SF pain scores) will be compared. Use of breakthrough medication (during treatment) will be recorded. In addition, the change from baseline in mean number of doses of escape medication will be analyzed using ANCOVA.

10.4 Interim Analyses

If Stage 1 PK data is available an interim analysis will be conducted to evaluate the rate and extent to which the drug's active ingredients namely THC and CBD and other secondary cannabinoids are made available to the body and the way they are metabolized in this stage of the study. However the extent of cannabinoid organ distribution and excretion data will not be possible.

11 DATA MANAGEMENT

11.1 Data Collection

Clinical trial data acquisition will be according to the CRF and will follow the data flow from the perspective of the investigator completing the CRF. This process will take into account the flow of study procedures and typical organization of data in a medical record.

11.2 Data Storage

Clinical case reports will be manually completed and stored in locked file cabinets at the study site.
CRF data transfer to an electronic database and will be securely maintained on an electronic disk that is password protected. This dataset will be located and maintained at Write Source Medical with the independent statistician (Dr Belinda Butcher), at PO Box 1521| Lane Cove NSW 2066.

11.3 Data Confidentiality

In order to protect participant information privacy, all data will be de-identified and will be recorded with a unique identifier and as such will be re-identifiable. Furthermore, note that the master list containing participant identifiers will remain at the study site.

11.4 Study Record Retention

This study will incur a moderate risk to participants and as such the files will be retained in a suitable repository for a minimum of 15 years and will be funded by the sponsor.

12 ADMINISTRATIVE ASPECTS

Prior to participant enrolment the clinical trial will be registered with the Australia New Zealand Clinical Trials Registry – ANZCTR. Reference number will be provided.

12.1 Independent HREC approval

Prior to participant enrolment the clinical trial will have been approved, by the Northern Sydney Local Health District HREC. Reference number will be provided.

12.2 Amendments to the protocol

Any amendments will be submitted to the HREC for review prior to implementation as per HREC guidelines.

12.3 Protocol deviations

Any protocol deviations will be submitted to the HREC for review.

12.4 Participant reimbursement

Participants will be reimbursed for parking and meals on trial days at study site.

12.5Financial disclosure and conflicts of interest

- Medlab Clinical Ltd. is the sponsor of the study and the entity involved in developing the cannabis medicine.
- Professor Luis Vitetta (AI5) and Mr Sean Hall (AI4) are employees of Medlab Clinical Ltd.

13 USE OF DATA AND PUBLICATIONS POLICY

This study will most likely be the subject of invited plenary presentations at national and international meetings and will likely result in influential publications. Participant privacy will be maintained as no participant will be individually identified in any publication.

14 REFERENCES

1. Flach S, et al. A Phase I Study to Investigate the Absorption, Pharmacokinetics, and Excretion of [14C] Prucalopride After a Single Oral Dose in Healthy Volunteers. Clinical Therapeutics 2016;38(9):2106-2115.
2. Ando Y, Inada-Inoue M, Mitsuma A, et al. Phase I dose-escalation study of buparlisib (BKM120), an oral pan-class I PI3K inhibitor, in Japanese patients with advanced solid tumors. Cancer Science 2014;105(3):347-353.
3. Rog DJ, Nurmikko TJ, Friede T, et al. Randomized, controlled trial of cannabis-based medicine in central pain in multiple sclerosis. Neurology 2005;65:812**e**819.
4. Berman JS, Symonds C, Birch R. Efficacy of two cannabis based medicinal extracts for relief of central neuropathic pain from brachial plexus avulsion: results of a randomised controlled trial. Pain 2004;112:299**e**306
5. Hochberg Y. A sharper Bonferroni procedure for multiple tests of significance. Biometrika 1988;75:800**e**802.
6. Conover WJ, Inam RL. Analysis of covariance using the rank transformation. Biometrics 1982;38:715**e**724.
7. Hodges JL Jr, Lehmann EL. Estimates of location based on rank tests. Ann Math Statist 1963;34:598**e**611.
8. The National Statement on Ethical Conduct in Human Research (NHMRC 2007) (National Statement). (http://www.nhmrc.gov.au/publications/synopses/e72syn.htm)
9. Note for Guidance on Good Clinical Practice (CPMP/ICH/135/95) - annotated with TGA comments. DSEB. July 2000. (http://www.tga.gov.au/docs/html/ich13595.htm)
10. Note for Guidance on Clinical Safety Data Management: Definitions and Standards for Expedited Reporting (CPMP/ICH/377/95). Annotated with TGA comments, August 2001. (http://www.tga.gov.au/docs/html/ich37795.htm)
11. Kramer JL. Medical Marijuana for Cancer CA Cancer J Clin 2015;65:109-122.
12. Brenneisen R. Chemistry and analysis of phytocannabinoids and other cannabis constituents. In: Elsohly M, ed. Marijuana and the Cannabinoids. Totowa, NJ: Human Press; 2007:17-51.
13. Pertwee RG. The diverse CB1 and CB2 receptor pharmacology of three plant cannabinoids: delta9-tetrahydrocannabinol, cannabidiol and delta9-tetrahydrocannabivarin. Br J Pharmacol 2008;153:199-215.
14. Rog DJ, Nurmikko TJ, Friede T, et al. Randomized, controlled trial of cannabis-based medicine in central pain in multiple sclerosis. Neurology 2005;65:812**e**819.
15. Berman JS, Symonds C, Birch R. Efficacy of two cannabis based medicinal extracts for relief of central neuropathic pain from brachial plexus avulsion: results of a randomised controlled trial. Pain 2004;112:299**e**306.
16. Bhattacharyya S, Morrison PD, Fusar-Poli P, et al. Opposite effects of delta-9-tetrahydrocannabinol and cannabidiol on human brain function and psychopathology. Neuropsychopharmacology 2010;35:764-774.
17. Grotenhermen F. Pharmacokinetics and pharmacodynamics of cannabinoids. Clin Pharmacokinet 2003;42:327-360.
18. Ohlsson A, Lindgren JE,Wahlen A, et al. Plasma delta-9 tetrahydrocannabinol concentrations and clinical effects after oral and intravenous administration and smoking. Clin Pharmacol Ther 1980;28:409-416.
19. Guy GW, Flint ME. A single centre, placebo-controlled, four period, crossover, tolerability study assessing pharmacodynamics effects, pharmacokinetic characteristics and cognitive profiles of a single dose of three formulations of cannabis based medicine extracts (CBMEs) (GWPD9901), plus a two period tolerability study comparing pharmacodynamic effects and pharmacokinetic characteristics of a single dose of a cannabis based medicine extract given via two administration routes (GWPD9901 EXT). J Cannabis Ther 2003 (3/4):35-77.
20. Karschner EL, Darwin WD, Goodwin RS, Wright S, Huestis MA. Plasma cannabinoid pharmacokinetics following controlled oral delta9-tetrahydrocannabinol and oromucosal cannabis extract administration. Clin Chem 2011;57:66-75.
21. Stott CG, White L, Wright S, et al. A phase I study to assess the effect of food on the single dose bioavailability of the THC/CBD oromucosal spray. Eur J Clin Pharmacol 2013;69:825-834.
22. Mechoulam R, Parker LA, Gallily R. Cannabidiol: an overview of some pharmacological aspects. J Clin

Pharmacol 2002;42(suppl 11):11S-19S.
23. Ware MA, Wang T, Shapiro S, et al. Smoked cannabis for chronic neuropathic pain: a randomized controlled trial. CMAJ 2010;182:E694-E701.
24. Ellis RJ, Toperoff W, Vaida F, et al. Smoked medicinal cannabis for neuropathic pain in HIV: a randomized, crossover clinical trial. Neuropsychopharmacology 2009;34:672-680.
25. Abrams DI, Jay CA, Shade SB, et al. Cannabis in painful HIV-associated sensory neuropathy: a randomized placebo controlled trial. Neurology 2007;68:515-521.
26. Wilsey B, Marcotte T, Deutsch R, Gouaux B, Sakai S, Donaghe H. Low-dose vaporized cannabis significantly improves neuropathic pain. J Pain 2013;14:136-148.
27. Abrams DI, Couey P, Shade SB, Kelly ME, Benowitz NL. Cannabinoid-opioid interaction in chronic pain. Clin Pharmacol Ther 2011;90:844-851.
28. Milstein SL, MacCannell K, Karr G, Clark S. Marijuana-produced changes in pain tolerance. Experienced and nonexperienced subjects. Int Pharmacopsychiatry. 1975;10:177-182.
29. Cooper ZD, Comer SD, Haney M. Comparison of the analgesic effects of dronabinol and smoked marijuana in daily marijuana smokers. Neuropsychopharmacology. 2013;38:1984-1992.
30. Greenwald MK, Stitzer ML. Antinociceptive, subjective and behavioral effects of smoked marijuana in humans. Drug Alcohol Depend 2000;59:261-275.
31. Wallace M, Schulteis G, Atkinson JH, et al. Dose-dependent effects of smoked cannabis on capsaicin-induced pain and hyperalgesia in healthy volunteers. Anesthesiology. 2007;107:785-796.
32. Ware MA, Wang T, Shapiro S, et al. Smoked cannabis for chronic neuropathic pain: a randomized controlled trial. CMAJ 2010;182:E694-E701.
33. Ellis RJ, Toperoff W, Vaida F, et al. Smoked medicinal cannabis for neuropathic pain in HIV: a randomized, crossover clinical trial. Neuropsychopharmacology. 2009;34:672-680.
34. Sutton IR, Daeninck P. Cannabinoids in the management of intractable chemotherapy-induced nausea and vomiting and cancer related pain. J Support Oncol 2006;**4**:531–535.
35. Guzman M. Cannabinoids: potential anticancer agents. Nat Rev Cancer 2003;3:745–755.
36. Kathmann M, Flau K, Redmer A, et al. Cannabidiol is an allosteric modulator at mu- and delta-opioid receptors. Naunyn Schmiedebergs Arch Pharmacol. 2006;372(5):354-61
37. Joy JE, Watson SJ, Benson JA. (eds). Marijuana and Medicine: Assessing the Science Base (National Academy Press, Washington, DC, 1999). Cited: Abrams DI, Guzman M. Cannabis in Cancer Care Clinical Pharmacology and Therapeutics 2015;97(6):575-586.
38. Huang YH. et al. An epidemiologic review of marijuana and cancer: an update. Cancer Epidemiol. Biomarkers Prev 2015;24:15–31.
39. TGA.[https://www.tga.gov.au/publication/access-unapproved-therapeutic-goods-clinical-trials-australia]

15 APPENDICES

APPENDIX 1:

THE QLQ-C30 VERSION 1.0 WITH FUNCTIONAL / SYMPTOM SCALES INDICATED

	SCALE	NO	YES
1. Do you have any trouble doing strenuous activities, like carrying a heavy shopping bag or a suitcase?	Physical	1	2
2. Do you have any trouble taking a **long** walk?	Physical	1	2
3. Do you have any trouble take a **short** walk outside of the house?	Physical	1	2
4. Do have to stay in bed or a chair for most of the day?	Physical	1	2
5. Do you need help with eating, dressing, washing yourself or using the toilet?	Physical	1	2
6. Are you limited in any way in doing either your work or doing household jobs?	Role	1	2
7. Are you completely unable to work at a job or to do household jobs?	Role	1	2

**During the past week:**	SCALE	**Not at all**	**A little**	**Quite a bit**	**Very much**
8. Were you short of breath?	Dyspnoea	1	2	3	4
9. Have you had pain?	Pain	1	2	3	4
10. Did you need rest?	Fatigue	1	2	3	4
11. Have you had trouble sleeping?	Insomnia	1	2	3	4
12. Have you felt weak?	Fatigue	1	2	3	4
13. Have you lacked appetite?	Appetite Loss	1	2	3	4
14. Have you felt nauseated?	Nausea and Vomiting	1	2	3	4
15. Have you vomited?	Nausea and Vomiting	1	2	3	4

**During the past week:**	SCALE	**Not at all**	**A little**	**Quite a bit**	**Very much**
16. Have you been constipated?	Constipation	1	2	3	4

17. Have you had diarrhoea?	Diarrhoea	1	2	3	4
18. Were you tired?	Fatigue	1	2	3	4
19. Did pain interfere with you daily activities?	Pain	1	2	3	4
20. Have you had difficulty in concentrating on things, like reading a newspaper or watching television?	Cognitive	1	2	3	4
21. Did you feel tense?	Emotional	1	2	3	4
22. Did you worry?	Emotional	1	2	3	4
23. Did you feel irritable?	Emotional	1	2	3	4
24. Did you feel depressed?	Emotional	1	2	3	4
25. Have you had difficulty remembering things?	Cognitive	1	2	3	4
26. Has your physical condition or medical treatment interfered with your **family** life?	Social	1	2	3	4
27. Has your physical condition or medical treatment interfered with your **social** activities?	Social	1	2	3	4
28. Has your physical condition or medical treatment caused you financial difficulties?	Financial Difficulties	1	2	3	4

GLOBAL HEALTH STATUS

29. How would you rate your overall **physical condition** during the past week?

1<br>Very poor	2	3	4	5	6	7<br>Excellent

30. How would you rate your overall **quality of life** during the past week?

1<br>Very poor	2	3	4	5	6	7<br>Excellent

APPENDIX 2:

Numerical Pain Rating Scale

You may experience some pain from cancer or cancer treatment. Only you know how much pain you have. You need to be able to describe your pain to your health care team, as well as to your family or friends.

Describe How Much Pain You Feel

Using a pian rating scale, like the one below, is helpful in describing how much pain you are feeling.

No pain				Moderate pain					Worst pain	
0	1	2	3	4	5	6	7	8	9	10

Try to assign a number from 0 (zero) to 10 (ten) to your pain level. If you have no pain, use 0. As the numbers get higher, they stand for pain that is getting worse. A 10 means the pain is as bad as it can be.

You can use a rating scale to describe:

how your pain feels at its worst.
how your pain feels most of the time.
how your pain feels at its least.
how your pain changes with treatment.

APPENDIX 3:

Brief Pain Inventory – Short Form

Patient name: ______________________ **Date:** __________

1 Throughout our lives, most of us have had pain from time to time (such as minor headaches, sprains, and toothaches). Have you had pain other than these everyday kinds of pain today?	**Yes** ☐	**No** ☐

2 On the diagram, shade in the areas where you feel pain. Put an X on the area that hurts the most.

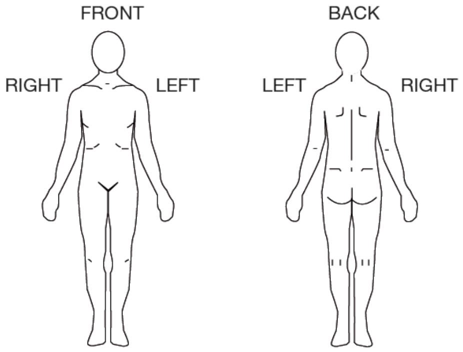

Comments:

3 Please rate your pain by selecting the one number that best describes your pain at its WORST in the past 24 hours.

**No pain**										**Pain as bad as you can imagine**
0	1	2	3	4	5	6	7	8	9	10
○	○	○	○	○	○	○	○	○	○	○

4 Please rate your pain by selecting the one number that best describes your pain at its LEAST in the past 24 hours.

**No pain**										**Pain as bad as you can imagine**
0	1	2	3	4	5	6	7	8	9	10
○	○	○	○	○	○	○	○	○	○	○

5 Please rate your pain by selecting the one number that best describes your pain on AVERAGE.

**No pain**										**Pain as bad as you can imagine**
0	1	2	3	4	5	6	7	8	9	10
○	○	○	○	○	○	○	○	○	○	○

6 Please rate your pain by selecting the one number that tells how much pain you have RIGHT NOW.

**No pain**										**Pain as bad as you can imagine**
0	1	2	3	4	5	6	7	8	9	10
○	○	○	○	○	○	○	○	○	○	○

7 What treatments or medications are you receiving for your pain?

8 In the past 24 hours, how much RELIEF have pain treatments or medications provided: Please select the one percentage that most shows how much.

**No relief**										**Complete relief**
0%	10%	20%	30%	40%	50%	60%	70%	80%	90%	100%
○	○	○	○	○	○	○	○	○	○	○

Brief Pain Inventory continues overleaf ▸
